# Different methods of synthesizing poly(glycerol sebacate) (PGS): A review

**DOI:** 10.3389/fbioe.2022.1033827

**Published:** 2022-11-30

**Authors:** Bruno Godinho, Nuno Gama, Artur Ferreira

**Affiliations:** ^1^ CICECO-Aveiro Institute of Materials, University of Aveiro, Aveiro, Portugal; ^2^ ESTGA-Águeda School of Technology and Management, Águeda, Portugal

**Keywords:** poly(glycerol sebacate) (PGS), microwave-assisted synthesis, enzymatic synthesis, polycondensation synthesis, PGS-based materials

## Abstract

Poly(glycerol sebacate) (PGS) is a biodegradable elastomer that has attracted increasing attention as a potential material for applications in biological tissue engineering. The conventional method of synthesis, first described in 2002, is based on the polycondensation of glycerol and sebacic acid, but it is a time-consuming and energy-intensive process. In recent years, new approaches for producing PGS, PGS blends, and PGS copolymers have been reported to not only reduce the time and energy required to obtain the final material but also to adjust the properties and processability of the PGS-based materials based on the desired applications. This review compiles more than 20 years of PGS synthesis reports, reported inconsistencies, and proposed alternatives to more rapidly produce PGS polymer structures or PGS derivatives with tailor-made properties. Synthesis conditions such as temperature, reaction time, reagent ratio, atmosphere, catalysts, microwave-assisted synthesis, and PGS modifications (urethane and acrylate groups, blends, and copolymers) were revisited to present and discuss the diverse alternatives to produce and adapt PGS.

## 1 Introduction of poly (glycerol sebacate) (PGS)

**FIGURE 1:**
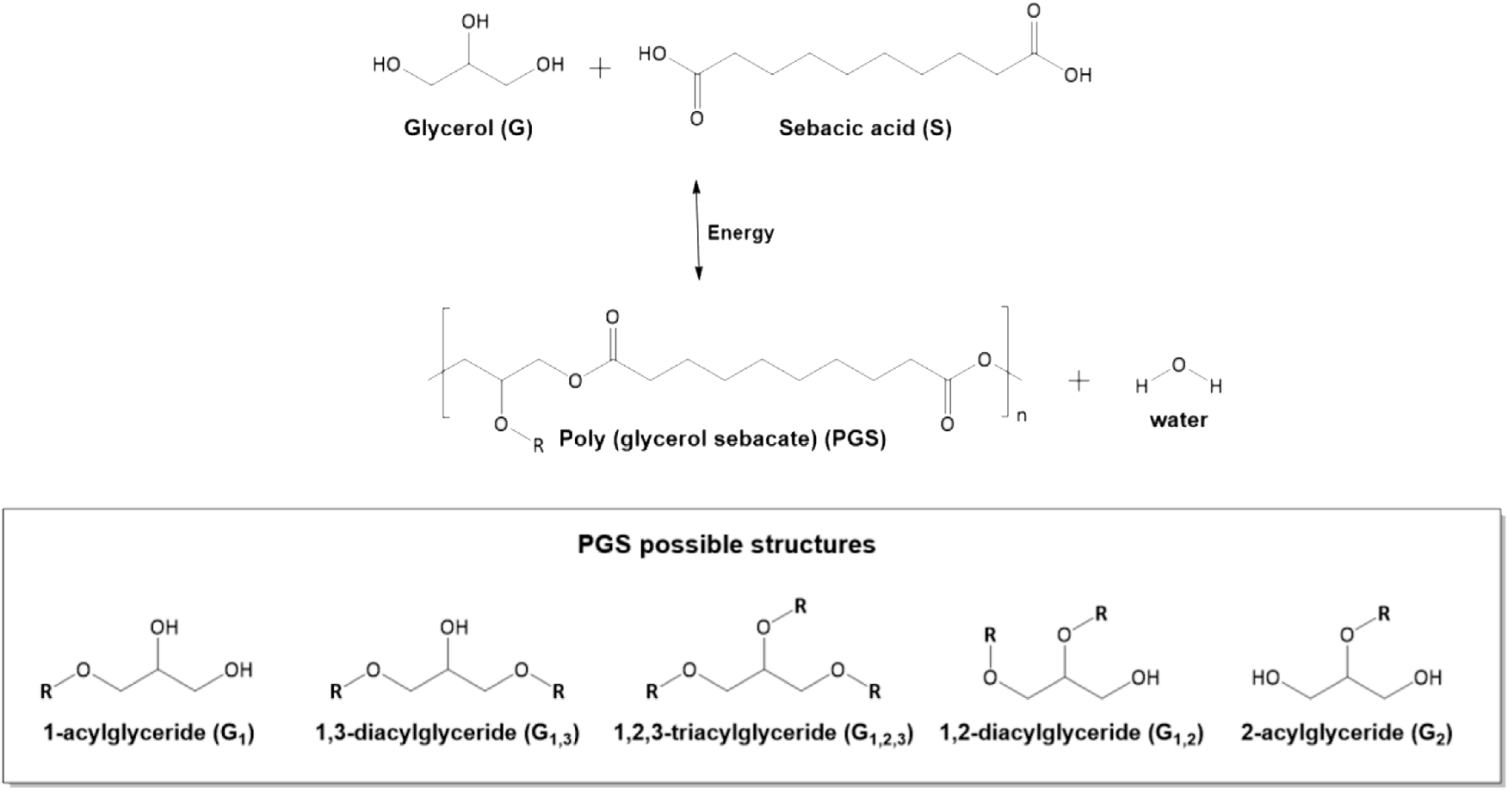
Schematic of the polycondensation of glycerol and sebacic acid to produce poly(glycerol sebacate) (PGS) and possible structures in the polymer chains. “R” = undefined polymer chain.

Poly(glycerol sebacate) (PGS) is a polyester elastomer conventionally produced through the esterification of glycerol with sebacic acid (Figure 1). It is bioresorbable and biodegradable; moreover, its degradation results in non-toxic products. Since its first report as a biocompatible material in 2002 (Wang et al., 2002), PGS has been a research focus of many groups. However, publications before Wang et al. (2002) by Nagata et al. (1996; 1999) described the synthesis of a PGS film (considered a biodegradable polyester) via polycondensation, which was identified as “Yg10” rather than PGS (Nagata et al., 1996; Nagata et al., 1999). The absence of these terms may explain why they have been somewhat forgotten in the literature. Some review articles erroneously consider Wang et al. (2002) the first report of PGS synthesis (Loh et al., 2015; Halil Murat, 2017; Sha et al., 2021; Vogt et al., 2021; Wu et al., 2021).

Several studies have targeted the comprehension and optimization of PGS synthesis and its properties, which have analyzed numerous variables (Liu et al., 2007a; Liu et al., 2007b; Kossivas et al., 2012; Li et al., 2013a; Li et al., 2013b; Guo et al., 2014; Li et al., 2015; Moorhoff et al., 2015; Conejero-García et al., 2017; Gadomska-Gajadhur et al., 2018; Matyszczak et al., 2020; Perin and Felisberti, 2020; Vilariño-Feltrer et al., 2020; Martín-Cabezuelo et al., 2021a; Martín-Cabezuelo et al., 2021b; Wrzecionek et al., 2021; Ning et al., 2022). In addition to these fundamental studies, PGS has been used as a component of polymer blends (Frydrych et al., 2015a; Tallawi et al., 2016; Salehi et al., 2017; Gultekinoglu et al., 2019; Saudi et al., 2019; Fakhrali et al., 2020; Flaig et al., 2020; Gorgani et al., 2020; Kaya et al., 2020; Stowell et al., 2020; Xuan et al., 2020; Behtaj et al., 2021a; Behtaj et al., 2021b; Fakhari et al., 2021; Hanif et al., 2021; Mokhtari and Zargar Kharazi, 2021; Varshosaz et al., 2021; Zhang et al., 2021; Fakhrali et al., 2022), other composite materials (Redenti et al., 2009; Chen et al., 2010; Liang et al., 2011; Gaharwar et al., 2015; Zhou et al., 2015; Rosenbalm et al., 2016; Souza et al., 2017; Tevlek et al., 2017; Zhang et al., 2017; Chen S et al., 2018; Abudula et al., 2020; Fu et al., 2020a; Aghajan et al., 2020; Sencadas et al., 2020a; Lau et al., 2020; Luginina et al., 2020; Rezk et al., 2020; Ruther et al., 2020; Tallá Ferrer et al., 2020; Touré et al., 2020; Zanjanizadeh Ezazi et al., 2020; Piszko et al., 2021a; Atya et al., 2021; Fakhrali et al., 2021; Rastegar et al., 2021; Talebi et al., 2021; Wang et al., 2021; Davoodi et al., 2022), or chemically modified/integrated in the development of PGS copolymers (Tang et al., 2006; Wu Y et al., 2014; Aydin et al., 2016; Jia et al., 2016; Choi et al., 2017; Tang et al., 2017; Zhao et al., 2017; Wang et al., 2018; Wilson et al., 2018; Lang et al., 2020; Rostamian et al., 2020; Chang and Yeh, 2021; Azerêdo et al., 2022; Ruther et al., 2022). PGS-based polymers have been widely employed in electrospinning to produce fibers (Yi and La Van, 2008; Jeffries et al., 2015; Rai et al., 2015; Tallawi et al., 2015; Hou et al., 2017; Hu et al., 2017; Vogt et al., 2018; Wu H. J. et al., 2019; Saudi et al., 2019; Vogt et al., 2019; Abudula et al., 2020; Apsite et al., 2020; Fakhrali et al., 2020; Flaig et al., 2020; Gorgani et al., 2020; Jafari et al., 2020; Keirouz et al., 2020; Silva et al., 2020; Stowell et al., 2020; Touré et al., 2020; Abazari et al., 2021; Bellani et al., 2021; Fakhari et al., 2021; Flaig et al., 2021; Mokhtari and Zargar Kharazi, 2021; Varshosaz et al., 2021; Zhang et al., 2021; Behtouei et al., 2022; Heydari et al., 2022; Rekabgardan et al., 2022; Saudi et al., 2022) and also studied for 3D printing (Yeh et al., 2016; Yeh et al., 2017; Chen S et al., 2018; Pashneh-Tala et al., 2018; Kazemzadeh Farizhandi et al., 2020; Touré et al., 2020; Tsai et al., 2020; Liu et al., 2021; Ruther et al., 2022).

The major focus of PGS-inspired polymers is the development of scaffold material for biological tissue engineering (Wang et al., 2002; Gao et al., 2006; Gao et al., 2007; Jeong and Hollister, 2010; Kemppainen and Hollister, 2010; Masoumi et al., 2013; Khosravi et al., 2016; Ma et al., 2016; Zhang et al., 2016; Nadim et al., 2017; Hsu et al., 2018; Wu W et al., 2019; Hu et al., 2019; Jiang et al., 2019; Xiao et al., 2019; Abudula et al., 2020; Fu et al., 2020a; Apsite et al., 2020; Fu et al., 2020b; Sencadas et al., 2020b; Jafari et al., 2020; Keirouz et al., 2020; Liang et al., 2020; Martín-Pat et al., 2020; Pashneh-Tala et al., 2020; Xuan et al., 2020; Abazari et al., 2021; Behtaj et al., 2021a; Piszko et al., 2021a; Liu et al., 2021; Rastegar et al., 2021; Ângelo et al., 2021; Chen et al., 2022; Fukunishi et al., 2022; Saudi et al., 2022), although many other purposes have been identified for these materials. PGS-based materials have been investigated as drug delivery systems (Sun et al., 2009; Sun et al., 2013; Yang et al., 2017; Ayati Najafabadi et al., 2018; Desai et al., 2018; Oklu et al., 2018; Zhu et al., 2018; Rezk et al., 2020; Zanjanizadeh Ezazi et al., 2020; Sivanesan et al., 2021; Torabi et al., 2021; Heydari et al., 2022; Mehta et al., 2022), adhesives (Mahdavi et al., 2008; Tevlek et al., 2017; Azerêdo et al., 2022), sealants (Chen et al., 2011), coatings (Kim et al., 2014; Lin et al., 2015; Jiang et al., 2020; Zbinden et al., 2020; Zhang et al., 2020; Martín-Cabezuelo et al., 2021b; Ghafarzadeh et al., 2021), biosorbents (Rostamian et al., 2022), membranes for solvents/water pervaporation (Chang et al., 2021), and components for electronic applications (Chen S et al., 2018; Sencadas et al., 2020a; Kazemzadeh Farizhandi et al., 2020; Zhang et al., 2020; Hanif et al., 2021). Their memory shape properties have also been studied (Cai and Liu, 2007; Wu T et al., 2014; Rosenbalm et al., 2016; Wu et al., 2016; Coativy et al., 2017; Tevlek et al., 2020; Xuan et al., 2020). Several reviews have also compiled the research, developments, and applications of PGS and PGS-based materials (Rai et al., 2012; Loh et al., 2015; Halil Murat, 2017; Valerio et al., 2018; Piszko et al., 2021b; Sha et al., 2021; Vogt et al., 2021; Wu et al., 2021; Zulkifli et al., 2022).

The literature used for the present review was identified through searches of the Scopus, Web of Science, Google Scholar, and ResearchGate databases in the Mendeley^®^ application, which was also used to store the database of identified studies. Additionally, we thank the reviewers who also suggested relevant publications to enrich this review article. The search included the following keywords and phrases: “PGS,” “poly(glycerol sebacate),” “poly(glycerol-co-diacids),” “enzymatic synthesis polyesters,” “glycerol polyesters,” “microwave-assisted polyester synthesis,” “PGS-based materials,” etc.

We did not limit the search to any specific period; thus, this review includes relevant publications from 1996 up to 2022.

This review focused on the different synthesis routes used to produce PGS, including its variables and how they influence the polymer properties. The review begins with the conventional method for PGS synthesis *via* the polycondensation of glycerol with sebacic acid, followed by polycondensation at higher temperatures and microwave-assisted polycondensation. Next, the review describes enzymatic synthesis and the use of other monomers to obtain PGS. Under each of these topics, efforts were made to ensure that the review followed the chronological order of publications whenever possible. We believe that this chronological organization of the publications allows readers to better understand the origin and evolution of knowledge. The following sections present publications on the use of other catalysts and other monomers to obtain the polymer structure of PGS, cross-linking of PGS by photopolymerization, and urethane bonds. The review ends with the properties of PGS and PGS-based materials, where the degradative behavior of these materials is presented in detail (e.g., *in vitro*, *in vitro* enzymatic, and *in vivo*).

This review consolidates the PGS and PGS-based materials synthesis comprehension in their several variables and routes. Therefore, this is a tribute to the knowledge developed over more than 20 years of research, especially publications from the 1990s that were somewhat overlooked, and that also provided fundamental knowledge on the field of PGS.

## 2 PGS synthesis

### 2.1 PGS polycondensation synthesis (conventional method)

Traditional PGS synthesis is an energy-intensive and time-consuming process. However, it is considered an economic material (Li et al., 2013a; Kafouris et al., 2013). The conventional method involves a two-step procedure that incorporates a prepolymerization step to form low molecular weight polymers/oligomers, followed by a curing step to cross-link these products and shape the final material. Both steps are normally performed at around 120–150°C, under an inert atmosphere or vacuum, and without catalysts or solvents. Wang et al. (2002) proposed a procedure to produce PGS; subsequent works suggested changes or small adjustments based on those processes (Table 1).

**TABLE 1:** Synthesis conditions for PGS and PGS-based materials.

Application/Objective	Molar ratio G:S	Prepolymerization stage	Curing step	References
		The (number) means steps order		
Ancient article (year 1996) before the “PGS” expression	2:3	200°C, 2 h, nitrogen	Film cast (20 wt% DMF solution 80°C)	Nagata et al. (1996)
Properties assessment (time of cure effect)		PGS prepolymer (called Yg10 by the authors)	Aluminum plate mold	
			230°C, 30 min, 1, 2, 4, and 6 h, nitrogen	
			Transparent and flexible film of Yg10 (insoluble in organic solvents for polyesters)	
Ancient article (year 1999) before the “PGS” expression	2:3	200°C, 43 min, nitrogen	Film cast (17 wt% DMF solution 80°C)	Nagata et al. (1999)
Properties assessment (effect of sebacic acid progressive substitution by other diacids to produce PGS copolymers)		PGS prepolymer (called Yg10 by the authors)	Aluminum plate mold	
			230°C, 4 h, nitrogen	
			Transparent and flexible film of Yg10 (insoluble in organic solvents for polyesters)	
Wang et al. (2002) procedure (beginning of the “PGS” expression year 2002)	1:1	(1) 120°C, 24 h, argon	NaCl particles	Wang et al. (2002), Mitsak et al. (2012)
Porous scaffold for soft tissue engineering		(2) 120°C, 1 torr to 40 mTorr (5 h)	1,3-dioxilane	
		(3) 120°C., 48 h, 40 mTorr	PTFE mold	
			120°C, 100 mTorr	
Soft tissue engineering	1:1	(1) 120°C, 24 h, nitrogen	THF	Gao et al. (2006), Gao et al. (2007), Sales et al. (2007), Jeong and Hollister. (2010)
		(2) 120°C., 24 h, 40 mTorr	Salt mold disk	
		Highly viscous liquid (pale yellow)	150°C, 48 h, 100 mTorr	
Soft tissue engineering (myocardial tissue PGS match properties)	1:1	(1) 110 or 120 or 130°C, 24 h, argon	Sheet forming mold	Chen et al. (2007)
		(2) 110 or 120 or 130°C, 1 torr to 50 mmHg (5 h)	110 or 120 or 130°C, 48 h, vacuum oven 50 mmHg	
		(3) 110 or 120 or 130°C, 48 h, 50 mTorr		
Soft tissue engineering (heart valve)	1:1	(1) 120°C, 24 h, nitrogen	Sucrose coated glass microscope slides (mold) with prepolymer spread uniformly	Masoumi et al. (2013)
		(2) 120°C, 24 h, high vacuum (<50 mTorr)	120°C, 8, 12 or 16 h, high vacuum (<50 mTorr)	
		Viscous PGS prepolymer (hot)	Thin PGS membranes (∼250 nm)	
		Soft waxy prepolymer (room temperature)		
Cardiac tissue engineering	1:1	(1) 120°C, 24 h, nitrogen	Prepolymer “spinned” to fibers produce	Ravichandran et al. (2012)
		(2) 120°C., 48 h, 40 mTorr	130°C, 24 h	
		PGS prepolymer	PGS fibers	
Cardiac support devices	1:1	(1) 120°C, 24 h, nitrogen	120°C, 2 or 3 days, under vacuum	Chen et al. (2010)
		(2) Prepolymer mixed with nanoBioglass^®^ at 50°C	Think sheets (0.2–0.3 mm) PGS–Bioglass^®^ composites	
Scaffolds for skin tissue engineering	1:1	(1) 150°C, 12 h, nitrogen	Teflon circular mold	Zhang et al. (2016)
		(2) 150°C, 12 h, vacuum	140°C, 8, 9, 10, 12 or 13 h, vacuum oven	
		Highly viscous prepolymer (pale yellow)		
3D scaffolds for cartilage	4:3	(1) 120°C, 24 h, nitrogen	Teflon/hydroxyapatite mold	Kemppainen and Hollister (2010)
	1:1	(2) 120°C., 48 h, 50 mTorr	150°C, 24, 48 or 72 h, 100 mTorr	
	3:4			
Scaffolds for adipose tissue engineering	1:1	120°C, 72 h, nitrogen low flow	Prepolymer heated at 80°C and distributed in Teflon molds	Frydrych et al. (2015a)
		PGS prepolymer	Degassed film, 80°C vacuum oven, until void-free film	
			120°C, 36 h, vacuum oven	
			PGS to blend with poly(lactic acid) PGS/PLA	
Neural tissue engineering	1:0.8	170°C, 3, 5 or 7 h, nitrogen	Prepolymer blended with poly(vinyl alcohol)	Saudi et al. (2019)
		PGS prepolymer	Electrospining of fibers (pPGS/PVA)	
			120°C, 24 h, 60 mmHg pressure	
			PGS-based fibers	
Nerve guide material	1:1	(1) 120°C, 24 h, argon	THF film cast	Sundback et al. (2005)
		(2) 120°C., 48 h, 40 mTorr	Dishes films	
		Viscous PGS prepolymer	120°C, 24 h, 40 mTorr	
Musculoskeletal tissue engineering	1:1	(1) 130°C, 2 h, argon	THF/prepolymer solution mixed with carbon nanotubes (CNTs)	Gaharwar et al. (2015)
		(2) 130°C, 1 torr to 50 mTorr (5 h)	Teflon flat petri dish. (THF evaporated overnight	
		(3) 120°C, 24 h, 50 mTorr	130°C, 40 h, vacuum	
		Prepolymer (Mw: 3960 g/mol)	PGS/CNTs nanocomposite scaffolds	
Sterilization effects and cytotoxicity and soft tissue engineering (cardiac patch)	1:1	120°C, 24 h, nitrogen	Teflon molds	Rai et al. (2013a), Rai et al. (2013b)
		Transparent viscous liquid prepolymer	120°C, 4 days, under vacuum (1.3–2.5 × 10^–2^ mbar) for 4 days	
			PGS transparent film (1.5 mm)	
Scaffolds to restoring a wounded rat uterus	1:1	(1) 120°C, 24 h, nitrogen	Solvent cast and particles leaching (THF and NaCl)	Xiao et al. (2019)
		(2) 120°C, 24 h, 1 torr	Disciform mold	
		PGS prepolymer	150°C, 24 h, 1 torr	
Properties assessment (thermoplastic and thermoset PGS)	2:2.5	(1) 130°C, 1 kPa, nitrogen (2:2)	Hot-pressed, 130°C, 15 MPa	Liu et al. (2007a), Liu et al. (2007b)
		(2) Sebacic acid (0.5) addition	Cold-pressed and molded, room °C, 20min	
		(3) 130°C, 1 kPa, nitrogen	Thermoplastic (TM)PGS	
Properties assessment (temperature of cure effect)	1:1	(1) 120°C, 24 h, nitrogen	120, 130, 140, 150 or 165°C, 24 h, vacuum oven (−20 kPa) or	Jaafar et al. (2010)
		(2) 120°C., 48 h, −20 kPa (instantly)	165°C, 2, 4, 10 or 48 h, same vacuum conditions	
Properties assessment (molar ratio effect)	2:1	(1) 120°C, 24 h, dry argon	Prepolymer transfer to mold at 120°C	Kossivas et al. (2012), Kafouris et al. (2013)
	2:2	(2) 120°C, 48 h, under vacuum	120°C, 24 h, under vacuum	
	2:3	Viscous branched PGS prepolymer		
	2:4	Prepolymer properties assessment		
	2:5			
Properties assessment (ratio G:S optimization for cell culture)	1:0.8	(1) 180°C, 2.5 h, nitrogen	NaCl mix with prepolymer	Guo et al. (2014)
	1:1	(2) 180°C, 1 h, vacuum	150°C, 24 h, vacuum	
	1:1.2			
Properties assessment	1:1	130°C, 24 h, nitrogen (130 cm^3^min^−1^ flow)	THF solvent	Li et al. (2013a), Li et al. (2013b), Moorhoff et al. (2015)
		Or	Film cast PGS prepolymer in glass slide molds	
		150°C, 8 h, nitrogen (130 cm^3^min^−1^ flow)	130°C, 24, 48, 72, 96, 144 or 168 h, under vacuum	
		PGS prepolymer	PGS gel sheets (0.5–0.9 mm)	
Synthesis for tailored mechanical properties	1:1	(1) Mix the two reagents at room temperature	Prepolymer/THF solution cast to aluminum mold	Li et al. (2015)
		(2) 120, 130 or 140°C, 24 h, convection oven under nitrogen (no agitation)	120, 130 or 140°C, 6–66 h, vacuum oven	
		Waxy or liquid like prepolymers (room temperature)		
Assessments for correlating properties with synthesis parameters of PGS.	2:1	130°C, 24 h, nitrogen	Teflon square mold	Conejero-García et al. (2017)
	1:1	Viscous PGS prepolymer	130°C, 24, 48, 72 or 96 h, ventilated oven or	
	1:2		110, 120, 140, 150°C, 48 h, ventilated oven	
Optimization synthesis of PGS for biomedical purposes (maximization aims degree of esterification and conversion of monomers)	1:1	(1) 130, 140, and 150°C as temperature variables and 4, 5 and 6 h as time reaction variables, under argon atmosphere	—	Gadomska-Gajadhur et al. (2018)
	2:1	(2) Distillation, 40°C, 18 mbar		
	3:1	(3) Purification		
		Dioxane/prepolymer solution, 24 h mixing		
		Cold distilled water addition for precipitation		
		Filtration and desiccation of PGS at 45°C, 24 h		
		Pure PGS prepolymer		
Material evaluation properties	1:1	(1) 130°C, 24 h, nitrogen	Teflon mold, ethanol evaporated at 60°C	Zhou et al. (2015)
		(2) Prepolymer/ethanol solution mixed with cellulose nanocrystals (CNC) at room temperature	130°C, 48 h, under vacuum	
			PGS/CNCs composite	
Drug carrier	1:1	150°C, 4 h, nitrogen	150°C, 30 h, under vacuum	Sun et al. (2009)
		Addition of 5-fluorouracil drug	PGS wafers 1–1.5 mm thickness	
Drug release (brain gliomas)	1:1.2	(1) 170°C, 1 h, nitrogen	170°C, 24 h, vacuum drying chamber	Sun et al. (2013)
		(2) 170°C, pressure reduce slow until vacuum, end after no bubble occurred	PGS polymer	
		PGS prepolymer	or	
		Or	Same procedure, but at 185°C	
		(1) (2) Same procedure, but 185°C	PGS-curcumin polymer	
		PGS-curcumin prepolymer		
Local drug delivery	1:1	(1) Evenly mixed reagents at room temperature	120°C, 72 h, vacuum oven	Yang et al. (2017)
		(2) 120°C, 24 h, nitrogen in vacuum oven (no agitation)	PGS drug load	
Memory shape material	1:1	(1) 120°C, 24 h, argon	THF	Cai and Liu, (2007)
		(2) 120°C., 48 h, 0.1 MPa	Films mold	
		Viscous PGS prepolymer	120°C, 24 h, 0.1 MPa	
			PGS films 1 mm thickness	
Memory shape material	1:1	(1) 120°C, 8 h, nitrogen	Prepolymer reacted with HDI to produce PGS urethane (PGSU) and mix with cellulose nanocomposites	Wu T et al. (2014)
		(2) 120°C, 16 h, vacuum oven		
		Viscous prepolymer		
Coating	1:1	(1) 130°C, 3 h, argon	Electrospray coating of nitinol stent with PGS prepolymer	Kim et al. (2014)
		(2) 120°C, 45 h, 40 mTorr	100°C, 48 h, vacuum oven	
		(Yield for viscous liquid phase prepolymer, above 80%)		
Assessments material for tissue engineering	1:1	(1) 130°C, 2 h, argon	Teflon crucibles mold	Patel et al. (2013)
		(2) 130°C, 1 torr to 40 mTorr (5 h)	130°C, 48 h, vacuum oven	
		(3) 130°C., 48 h, 40 mTorr	PGS polymer to copolymerize with poly(ethylene glycol)	
		PGS prepolymer		

Studies on the reaction kinetics have shown that the activation energy decreases with increasing molar ratios of glycerol to sebacic acid, which indicates that the reaction is favored at an equimolar ratio of reactants. The reaction kinetics also increase with increasing temperature, showing classical Arrhenius behavior (Maliger et al., 2013; Matyszczak et al., 2020).

Initially, the kinetic control of the reaction advances with first-order kinetics with respect to the monomer. When a given conversion is achieved, the viscosity of the medium increases by changing the reaction to a diffusion-controlled process, which makes it difficult to transfer mass in the system to continue the reaction (Valerio et al., 2018).

PGS properties can be modified by changing the reaction conditions (time, temperature, or reagent ratios) to produce a wide range of mechanical properties (Table 2). Liu et al. (2007a, 2007b) reported that PGS can be a thermoset (TS)PGS or a thermoplastic (TM)PGS depending on the molecular size of the prepolymer used to obtain cured PGS. This may influence the properties and final degradation rate of PGS. Different molecular weights cause the prepolymers to present different viscosities and reactivities in the curing step, which result in distinct branching degrees on the final products.

**TABLE 2:** Mechanical properties of PGS and PGS-based materials.

Material	Young’s modulus (MPa)	Tensile strength (MPa)	Elongation (%)	Compression strength (MPa)	References
PGS or PGS backbone type (PSeD)					
Poly(glycerol sebacate) (PGS)	0.017–6.86	0.1–1.96	10–448	2.75–4.74	Nagata et al. (1999), Wang et al. (2002), Chen et al. (2007), Mitsak et al. (2012), Patel et al. (2013), Gaharwar et al. (2015), Loh et al. (2015), Conejero-García et al. (2017), Tevlek et al. (2017), Wilson et al. (2018), Sencadas et al. (2020b), Lang et al. (2020), Tallá Ferrer et al. (2020), Atya et al. (2021)
				(54–70% deform)	
Poly(sebacoyl diglyceride) (PSeD)	1.57	1.83	409	—	You et al. (2010)
PGS copolymers					
Poly(glycerol glycol sebacate) PGGS	0.42–0.49	0.50–0.63	108–198	—	Tang et al. (2006)
PGS-co-poly(ethylene glycol) (PGS-co-PEG)	0.040–1.590	0.026–0.388	39–100%	1.88–2.99	Patel et al. (2013)
			(190% hydrated polymer)	(55–69% deform)	
Poly(glycerol sebacate citrate) (PGSC)	6.9	2.7	40	—	Liu et al. (2009)
PGS-co-lactic Acid (PGS-co-LA)	Sealant gel				Chen et al. (2011)
Poly(glycerol sebacate urethane) (PGSU)	0.1–20	0.14–12.1	78–516	0.13–0.75	Pereira et al. (2013), Wu T et al. (2014), Frydrych and Chen. (2017), Wang et al. (2018)
				(75% deform)	
				Scaffold material	
PGS-ureido-pyrimidinone-HDI (PGS-U)	0.4–32.8	0.2–4.6	610–260		Wu et al. (2016)
Urethane-based PEGylated PGS	1.0–6.4 (dry)	0.32–4.3 (dry)	53.6–272.8 (dry)		Wang et al. (2018)
	0.6–4.7 (hydrated)	0.14–3.7 (hydrated)	25.7–329.2 (hydrated)		
PGS-acrylate (PGSA)	0.05–30	0.01–1.36	5–200	—	Nijst et al. (2007), Ifkovits et al. (2008), Chen J.-Y et al. (2018)
PGS-poly(caprolactone) (PGS-PCL) fibers	5.6–15.7	2–3	142–900	—	Rai et al. (2015), Hou et al. (2017)
PGSA-co-polycaprolactone diacrylate (PGSA-co-PCLDA)	0.67–7	0.14–0.69	11.28–45.95	—	Chen S et al. (2018)
PGSA-co-poly(ethylene glycol) diacrylate (PGSA-co-PEGDA)	4.22–10.54	0.61–1.97	12.96–25.96	—	Chen S et al. (2018)
PGSA-co-PEGDA co-PCLDA	3.84–8.78	1.01–1.37	20.61–40.31	—	Chen S et al. (2018)
PGS-b-PTMO–Hytrel 3078	0.018	2.1	2574	—	Wilson et al. (2018)
Poly(glycerol-1,8-octanediol-sebacate) (PGOS)	106.1	4.94	23	—	Lang et al. (2020)
Palmitate-PGS (PPGS)	<0.3	<0.20	70–100	—	Fu et al. (2020b), Ding et al. (2020)
PGS-co-Zein	0.021–2.9	0.020–1.4	21–63	—	Ruther et al. (2022)
PGS-Citrate	0.12–1.29	±0.1–0.4	±30–120	—	Risley et al. (2021)
PGS composites/blends					
PGS/carbon nanotubes	0.28–1.01	0.13–0.275	38–99	—	Gaharwar et al. (2015)
(PGS-CNT) nanocomposites					
Multi-walled carbon nanotubes/(PGSC) (MWCNTs/PGSC)	0.85–9.9	0.9–4.4	40–325	—	Liu et al. (2009), Yan et al. (2018)
PLC/PGS/graphene	11.4–21.7	2.35–3.02	82.6–122.8	—	Fakhrali et al. (2021)
PGS/cellulose nanocrystals (PGS/CNCs)	1.0–1.9	0.62–1.5	80–100	—	Zhou et al. (2015)
PGSU/cellulose nanocrystals (PGSU/CNCs)	1.38–47.96	12.4	396	—	Wu T et al. (2014)
PGS/bacterial cellulose (PGS/BC)	1.21	0.32	25	—	Wang et al. (2021)
PGS/silk fibroin (PGS/SF)	1.5–2.5	1–6.5	100–325	—	Zhang et al. (2021)
PGS-β-tricalcium phosphate	1.95	0.21	24	14	Tevlek et al. (2017)
(PGS-β-TCP) bi-layered composites				(85% deform)	
(β-TCP/PGS content 15%) scaffolds	—	—	375	1.73	Yang et al. (2015)
PGS/Bioglass^®^	0.4–1.6	0.8–1.53	150–550	—	Chen et al. (2010), Rai et al. (2012)
PCL-PGS/bioactive glass	240–311	3–8	<5%	—	Touré et al. (2020)

Li et al. (2013a) identified inconsistencies in PGS properties among research groups using similar synthesis conditions. For example, Chen et al. (2007) and Jaafar et al. (2010) produced PGS with very different Young’s modulus of 1.2 and 0.12 MPa, respectively, despite identical reported synthesis conditions.

As previously stated, different synthesis conditions produce PGS with different properties. Chen et al. (2011) demonstrated some of these differences among research groups and proposed several explanations. For example, the temperature uniformity inside a vacuum oven is ±1°C at best and is typically ±2.5°C for most ovens (Chen et al., 2011). A difference of 5°C can significantly change the cross-linking kinetics of PGS, according to their experiences. Additionally, glycerol loss is problematic and inevitable. The purging flow rate with inert gas and the capacity of the vacuum pump can greatly affect glycerol loss during synthesis, altering the molar relationship of the reagents. Therefore, different research groups report different results, despite using apparently identical synthesis conditions (Chen et al., 2011).

Li et al. (2013a) confirmed that glycerol evaporation is the major cause of irreproducibility. This problem is aggravated when synthesis is performed at high temperatures. At the level of mechanical properties, Young’s modulus of PGS increases with longer cure duration and higher curing temperatures, while the ultimate strength at break decreased. The authors performed detailed ^1^H NMR and ^13^C NMR analyses. The results of the NMR analyses show that secondary hydroxyl groups, responsible for cross-link, reacted more slowly than primary hydroxyl groups. Thus, NMR techniques provided qualitative and semi-quantitative structural information on the synthesized PGS. The different structures of acylglycerides identified in PGS are shown in Figure 1.

**FIGURE 2:**
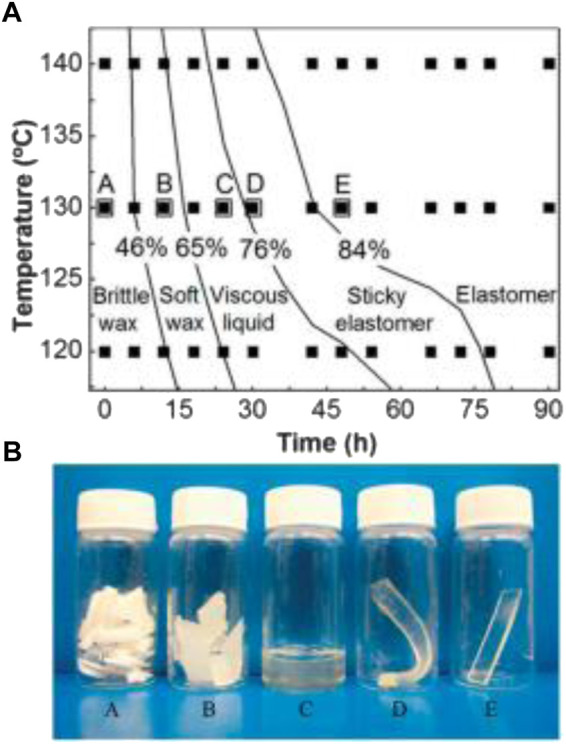
**(A)** Map showing the relationship between the degree of esterification and the state of the sample. The filled squares represent the time and temperature values for thermal treatment where the measurement of the degree of esterification and the physical state of each sample was examined. **(B)** Pictures of five samples at ambient temperature: A, brittle opaque wax; B, soft translucent wax; C, viscous translucent liquid; D, soft sticky elastomers; E, non-sticky elastomers. The five large squares with letters on top in **(A)** represent the thermal treatment conditions for the samples shown in **(B)**. Reproduced from Li et al. (2015).

Li et al. (2015) also identified glycerol loss as a problem. For example, the molar ratios of glycerol to sebacic acid decreased from 0.80 to 0.75 when the curing temperature increased from 120 to 140°C. The authors proposed the use of the degree of esterification to precisely predict the physical status, mechanical properties, and degradation of PGS. Young’s modulus linearly increased with the degree of esterification. Young’s modulus also increased as the total cure time increased, while the elongation at break decreased. Figure 2 illustrates the relationship between the degree of esterification (%) and the physical appearance of PGS at room temperature according to production time and temperature conditions (Li et al., 2015). Figure 2 shows the five physical states that can be observed at room temperature for the production of PGS at different times and temperatures. PGS starts in a waxy solid state, changing to a viscous liquid (gel state), and finally returning to a solid state.

Kafouris et al. (2013) also focused on understanding PGS synthesis by testing different molar ratios of G:S (glycerol:sebacic acid) to produce PGS (2:1, 2:2, 2:3, 2:4, and 2:5), using three reaction steps at 120°C (Table 1). All prepolymers were low molecular weight oligomers, between dimers and nonamers. They concluded that the properties of PGS elastomers are highly dependent on the composition. For example, PGS 2:3 elastomer was the stiffest, with the lowest degree of swelling and sol fraction, while the PGS 2:5 elastomer was one of the softest, exhibiting the highest degree of swelling and sol fraction. The elastomers with compositions far from stoichiometry were softer due to the lower cross-linking density.

In 2017, Conejero-García et al. (2017) assumed a disruptive approach face to the conventional method. It was the first work without vacuum steps and showed the second-highest Young’s modulus reported in the PGS literature (4.7 MPa). The prepolymerization step was conducted at 130°C under an inert atmosphere for 24 h. The curing step was performed in a forced ventilation oven. The resulting films were transparent, soft, and flexible, and became more yellowish and harder with increasing temperature or curing time. This yellowing may be related to the oxidation of the material due to the normal and ventilated atmosphere. The yellowing of polymeric materials due to oxidation is well known (Allen et al., 2022). PGS cured at low temperatures has more non-cross-linked chains and, thus, rinsing promotes a significant loss of mass. In comparison, curing at high temperatures results in more effective cross-linking. The curing time is also important in cross-linking, with mass losses ranging from 6% to 20% for PGS cured at 130°C/48 h and 130°C/24 h, respectively.

The ratio of reagents also influences the efficiency of cross-linking. Similar to Kafouris et al. (2013), Conejero-García et al. (2017) reported superior cross-link density at closer hydroxyl/carboxylic group equilibrium ratios, leading to more robust PGS.

Gadomska-Gajadhur et al. (2018) proposed an optimization of PGS prepolymer (pPGS) synthesis using the Box–Behnken design based on three variables (temperature, G:S molar ratio, and reaction time). The optimization criteria maximized the degree of esterification of PGS and the conversion of monomers. The optimal conditions resulted in a 2:1 molar ratio synthesis at 150°C for 5 h at reflux with stirring (200 rpm), argon atmosphere, and without a catalyst. The resulting pPGS showed a high conversion of the carboxylic groups (∼89%) and a very high degree of esterification (∼82%). The total process time to obtain pure material suitable for medical and pharmaceutical applications was >50 h (Table 1).

While the influences of temperature, time, and reagent molar ratio have been evaluated intensively, the effects of the atmosphere in the PGS reaction have been relatively neglected until recently.

In 2021, Martín-Cabezuelo et al. (2021a) reported the results of a study that aimed to better understand the effect of inert (argon and nitrogen) and oxidative (oxygen, dry air, and humid air) atmospheres in PGS synthesis. The prepolymerization step was performed at 130°C for 24 h with a 1:1 (G:S) molar ratio and different gases flowing through the reactor. The curing step was performed in an oven with forced ventilation at atmospheric pressure (130°C for 48 h). Synthesis at different atmosphere conditions led to PGS networks with significantly different properties. The prepolymerization step showed great extension when performed under oxidative atmospheres, but in a branched way due to the simultaneous formation of oxidized species that boost the reactivity of secondary hydroxyls from glycerol. In contrast, inert atmospheres (Ar even more than N_2_) promote linear growth of oligomers and low branching. As a result, the increase in viscosity was more gradual in pPGS obtained under inert atmospheres, so the gel point takes longer. After curing, PGS obtained from pPGS produced under oxidative atmospheres is less elastic and softer (Martín-Cabezuelo et al., 2021a).

### 2.2 PGS polycondensation synthesis (higher temperature approach)

The conventional method of PGS synthesis requires days to complete. To reduce PGS synthesis time, some research groups have used higher temperatures (≥170°C) (Sun et al., 2013; Guo et al., 2014; Gadomska-Gajadhur et al., 2018; Saudi et al., 2019; Riaz et al., 2022). This strategy is described in this section; some examples are also listed in Table 1.

Before the conventional method and the term “PGS” for this polymer were established, the first polycondensation of glycerol with sebacic acid and the production of PGS film (Yg10) were reported by Nagata et al. (1996 and 1999) The temperatures used for synthesis were extremely high and the researchers also used the two-step methodology. Prepolymerization and curing were performed at 200°C and 230°C, respectively, in both publications (Nagata et al., 1996; Nagata et al., 1999).

In 1996, Nagata et al. (1996) prepared aliphatic polyesters from glycerol and a series of various-length aliphatic dicarboxylic acids and analyzed the effects of the methylene chain length on the structure and physicochemical properties, as well as enzymatic degradation. The PGS film was prepared with various curing times (Table 1), which influenced the degree of reaction (%) and, consequently, the enzymatic degradation rate of the film. The PGS films with the best resistance to enzymatic degradation were produced with 2 h of prepolymerization followed by 2 h or 4 h of curing. At 6 h of curing time, the degree of reaction (%) was lower than that at 4 h. Therefore, 6 h was excessive, resulting in thermal degradation of the polymer (Nagata et al., 1996). This PGS film with 6 h of curing was also less resistant to enzymatic degradation.

In 1999, Nagata et al. (1999) prepared PGS copolymers by progressively replacing sebacic acid with other diacids and evaluated the physicochemical and thermal properties, as well as enzymatic degradation. The PGS film was obtained with 43 min of prepolymerization and 4 h of curing time. This very fast synthesis resulted in a material with 1.96 MPa of tensile strength, Young’s modulus of 6.86 MPa, and 27% elongation. Based on these values, this was the strongest and toughest PGS that we found in the literature.

Sun et al. (2013) were the first to report pPGS synthesis and cure at 170°C, significantly reducing the duration of this process. Furthermore, PGS-curcumin polymer was prepared at 185°C. Guo et al. (2014) also produced pPGS at 180°C in only 3.5 h (Table 1).

Matyszczak et al. (2020) created a kinetic model of the polycondensation of sebacic acid with glycerol based on infrared (IR) spectra during the reactions, which allowed the determination of the parameters of the Arrhenius equation over a wide temperature range (130°C–170°C). The polycondensation reaction was performed in an equimolar ratio of reactants at temperatures of 130°C, 150°C, and 170°C for 5–8 h, without any catalyst and under an argon atmosphere at 200 rpm. The disappearance of the 1410 cm^−1^ peak generated by the acid and an increasing intensity of the 1185 cm^−1^ ester peak were observed in the real-time IR measurement of the reactions. The polycondensation kinetics were determined based on changes in the intensity of these IR signals.

Saudi et al. (2019) synthesized pPGS under nitrogen gas at 170°C. Glycerol and sebacic acid were combined in a 1:0.8 (G:S) molar ratio as this ratio is more hydrophilic and suitable for cell adhesion and proliferation than other ratios (Guo et al., 2014). Under these conditions, the authors tested three reaction times (3, 5, and 7 h) and analyzed pPGS by Fourier transforming infrared (FTIR). They observed that, beyond 3 h, the sharp carbonyl peak at 1733 cm^−1^ shifted to 1691 cm^−1^ and its intensity increased. The sharp peak observed at 1691 cm^−1^ was related to carbonyl stretching of the unreacted free sebacic acid. Under these conditions, with increasing reaction time, the ester bonds were broken or degraded and a greater proportion of free sebacic acid remained after prepolymer formation. Based on these observations and compared to FTIR of the conventional pPGS synthesis in other studies, the authors suggested 3 h as the ideal reaction time.

The FTIR observations of Saudi et al. (2019) are contrary to the real-time IR spectra of pPGS synthesis reported by Matyszczak et al. (2020). At 5 h, the IR spectra showed a more intense peak related to ester groups compared to that at 3 h. For similar reaction conditions (only a slight ratio change of G:S, 1:0.8 and 1:1), the progression of reactions differed significantly between these studies (Saudi et al., 2019; Matyszczak et al., 2020).

Riaz et al. (2022) synthesized pPGS, mixing the reagents thoroughly for 15 min to ensure homogeneity, heating the reaction mixture at 180°C for 3 h, under continuous nitrogen flow, for use as an ultrasound contrast agent.

Synthesis by the conventional method can take several days without polymer degradation. At very high temperatures, polymer degradation can occur within hours, as reported by Nagata et al. (1996) and Saudi et al. (2019). These higher temperatures can also lead to a severe loss of glycerol. The temperature increase rate must be slow for the monomers to react, forming small monoglycerides (less volatile than glycerol) before reaching high temperatures. Users of this methodology should select the reaction time with care.

### 2.3 PGS microwave-assisted synthesis

Microwave-assisted synthesis (MwAS) is a time- and energy-efficient pathway to polycondensation reactions. The production of polyesters using microwaves is a relatively solid technology, with application on a non-laboratory scale. For example, in 2009, the first commercial plant for the mass production of poly(lactic acid) *via* microwave method was developed in Japan (Aydin et al., 2013).

MwAS significantly increases the esterification reaction rate by generating heat homogeneously in a bulk solution *via* dipole rotation, in which the polar species (e.g., glycerol and lactic acid) align themselves with a rapidly changing electric field produced by the microwaves such that the reactants can be activated selectively. Microwave irradiation provides heat internally and tends to eliminate the “thermal wall effect.” Hence, the condensed water molecules are evaporated faster in the microwave due to their large dielectric constant, which further enhances the polymerization reaction (Coativy et al., 2016; Lau et al., 2017).

Aydin et al. (2013) reported the first attempt to produce PGS using MwAS. The authors proposed an alternative for the initial prepolymerization step in 3 min instead of days, without purge gas, catalyst, vacuum, and agitation. Curing was performed at 150°C and 5 Torr for different time periods. They achieved a PGS after 3 min of prepolymerization and 16 h of cure with Young’s modulus of 0.50 ± 0.02 MPa, tensile strength of 0.27 ± 0.06 MPa, and an elongation of approximately 180%.

However, this MwAS reaction produced a polymer with a molar ratio different from the initial molar composition (G:S, 1:1 to 0.22:0.78). The process resulted in a severe loss of glycerol due to the reaction temperature during the first prepolymerization step, which resulted in the boiling of glycerol monomers, as well as the higher curing temperatures in the second step. The authors also suggested that, since the boiling point of glycerol is 290°C, the decreased time required for polymerization was caused by extremely high temperatures (Aydin et al., 2013).

Li et al. (2015) also performed a MwAS synthesis for prepolymerization. They demonstrated that 15 min of microwave time was as efficient as the conventional prepolymerization method in a nitrogen atmosphere for 6 h at 130°C. However, this rapid synthesis method causes severe glycerol evaporation, resulting in a large alteration in the ratio of the monomers, leading to a more rigid PGS produced under similar curing conditions compared to the conventional prepolymerization method. The temperature of the mixture in the microwave heating process reached 170°C. The glycerol loss was 63% after 30 min of microwave time. This value was significantly higher than the 5%–10% glycerol loss value for the samples prepolymerized for 24 h in a nitrogen atmosphere at 1 atm at 120–140°C.

**FIGURE 3:**
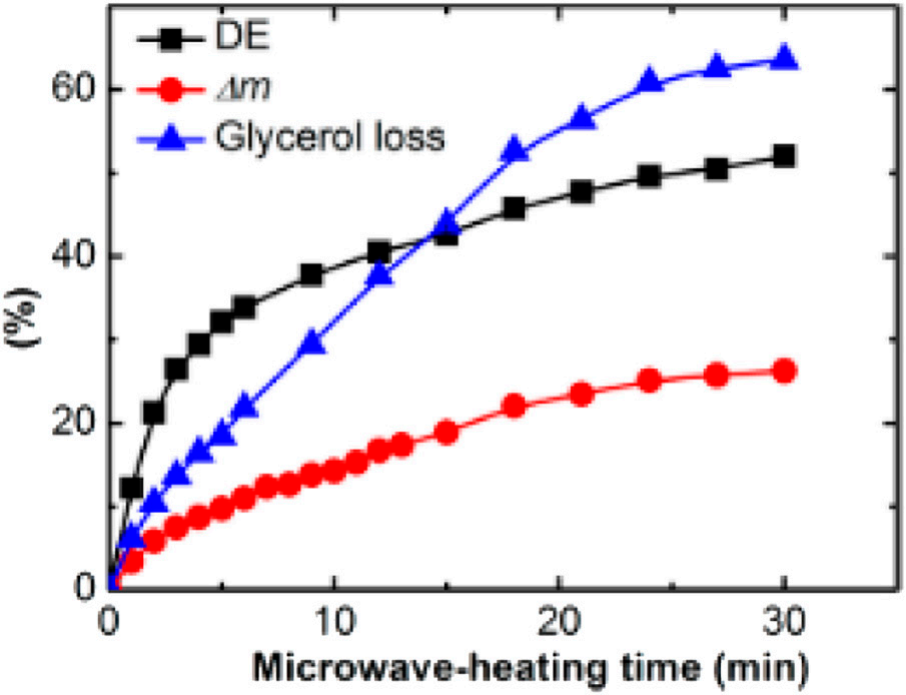
Evolution of the degree of esterification (DE), mass loss (Δ*m*), and glycerol loss values with increasing prepolymerization time during microwave heating (Li et al., 2015).

The results presented in Figure 3 (evolution of the degree of esterification (DE), mass loss (Δm), glycerol loss values, and prepolymerization time) indicate that the rate of esterification decreases faster after 3 min and show a severe loss of glycerol. Thus, the 3 min time is a trade-off between efficient prepolymerization and severe glycerol loss. The authors reported a PGS with Young’s modulus of 0.25 MPa, tensile strength of 0.25 MPa, and an elongation of approximately 190% for a microwave prepolymerization step lasting 3 min and a 48 h cure at 130°C in a vacuum, with a total loss of glycerol of 60%. The PGS properties are close to those reported by Aydin et al. (2013).

Tevlek et al. (2017) also produced pPGS in a microwave oven for 3 min at 650 W to mix with β-tricalcium phosphate (β-TCP) to create a bone-soft tissue interface. The mechanical properties of this material are shown in Table 2.

Recently, Tevlek et al. (2020) reported equimolar PGS with good elasticity (212.75 ± 37.25% elongation) and 0.09 ± 0.03 MPa (Young’s modulus) produced from microwave prepolymerization (4 min, with 10 s intervals every 1 min), followed by curing in a vacuum oven (150°C, 12 h).

Tevlek et al. (2022) also performed PGS prepolymerization in a microwave reactor (White-Westinghouse, United States) at 650 W without any catalyst or extra chemical material. The process was completed by exposing the reagents to microwaves for a total of five times, at 15 s intervals for 1 min. Curing was performed in a vacuum oven at 150°C and 10 mbar for 10, 12, and 14 h, to assess the impact of various cross-linking times on PGS membranes properties. More time (14 h) led to a PGS elastomer with more cross-link density, better biocompatibility, increased tensile strength, and lower elasticity.

Deniz et al. (2020) used a microwave oven (Samsung, Korea) at 650 W and high/medium settings. An equimolar sample of sebacic acid and glycerol mixture was exposed to five rounds of electromagnetic waves for 1 min each at 10-s intervals in the prepolymerization step.

**FIGURE 4:**
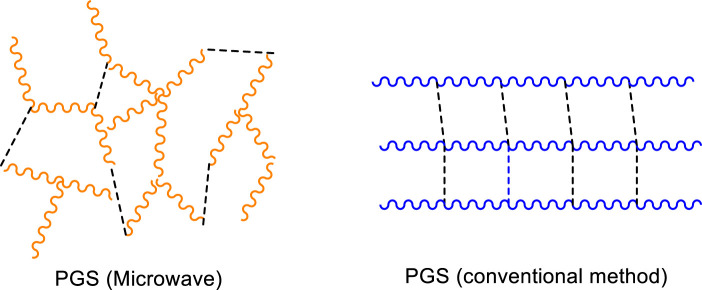
Proposed images of the possible structures of PGS prepolymerized *via* microwave and conventional methods. Dashed line: cross-linking. Images inspired by Lau et al. (2017).

Lau et al. (2017, 2020) proposed a solvent-based system (toluene) to provide better control of the reaction temperature in a microwave cavity and minimize monomer evaporation. Water was collected to measure the degree of esterification. The authors performed MwAS in a CEM Discover SP system, with the reaction maximum temperature well controlled at 130°C. This type of accuracy is impossible in conventional microwave ovens, similar to those used by Aydin et al. (2013) and Li et al. (2015). The curing step was performed at 120°C in a vacuum oven. MwAS was six times faster than conventional heating (CH). For example, 12 min of heating in MwAS, showed a DE of 66%; a similar value of CH required around 75 min. Furthermore, the results of NMR and MALDI-TOF analyses showed that the pPGS produced by MwAS was more branched than that produced by the conventional method without changing the molar ratio of glycerol and sebacic acid. Figure 4 shows proposed PGS structures by both methods. The microwave radiation interacts strongly with glycerol, leading to the activation of both alcohol groups (primary and secondary), which react more efficiently with sebacic acid compared to that in the CH approach.

The higher branching of the pPGS achieved by MwAS facilitates the formation of a cross-linked PGS in a very short curing time. For example, by reducing the curing time to 2 h, PGS specimens prepared by MwAS (DE = 66.82%) showed faster toughening compared to CH samples (DE = 68.18%). The PGS showed Young’s modulus values between 0.7 and 3.14 MPa and elongation between 60% and 15%, depending on curing time. A longer curing time resulted in higher Young’s modulus value and lower elasticity.

**FIGURE 5:**
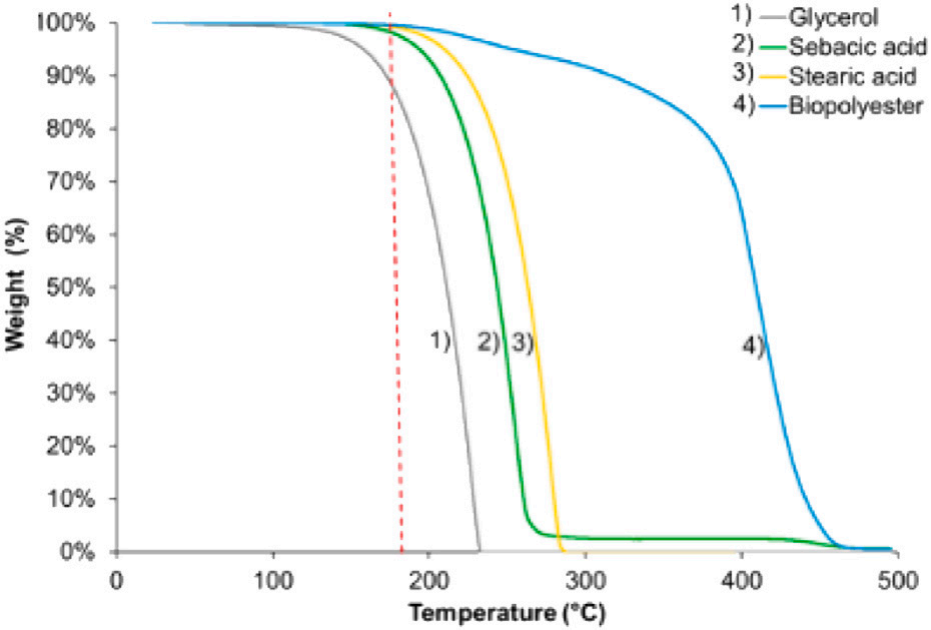
Thermogravimetric analysis: variation in reagent weight and biopolyesters under nitrogen flow with a heating ramp of 10°C/min (Coativy et al., 2016)

Coativy et al. (2016, 2017) also used a microwave approach to synthesize a modified PGS with stearic acid (a Microwave Synthesis Labstation MicroSYNTH with constant agitation). The reaction was performed at 180°C until the viscosity suddenly increased, resulting in the end of magnetic stirring. Stearic acid was used to limit cross-linking to adjust the original properties of PGS to produce a polymer with a memory shape (Coativy et al., 2017). This modified PGS was blended with PLA to increase its ductility (Coativy et al., 2016). Thermogravimetric analysis was performed on pure reagents and the formed polymer (Figure 5). These results were important to explain the glycerol loss behavior with temperature and gas flow. Glycerol showed two mass losses: a small 1% loss between room temperature and 150°C and a severe loss between 150°C and 220°C. This result highlights that glycerol, which is liquid, can be evaporated at a much lower temperature than its boiling point (290°C, 1 atm).

In addition to the boiling point, the flash point of a substance is also an important property to consider in evaporation processes. The flash point of glycerol is 160°C (Quispe et al., 2013), which confirms the observations of thermogravimetric analysis for the beginning of glycerol mass loss (Figure 5).

Lee et al. (2018) used a microwave reactor (Biotage^®^ Initiator, Charlotte, NC) to cure PGS. The microwave-cured PGS elastomers were similar to PGS elastomers produced by the conventional polycondensation method. The results showed that the microwave curing of PGS is feasible and eight times faster than the conventional curing process, with a maximum cross-link of PGS using a gradual heating up to 160°C for 3 h.

### 2.4 PGS enzymatic synthesis

Biosynthesis is an alternative to the conventional chemical process of polyester synthesis. Lipase-catalyzed polymerization has been widely investigated because it allows high catalytic activity and high selectivity at mild reaction conditions (preventing side reactions), without harmful components or metallic traces from inorganic catalysts. Enzymatic polymerization was demonstrated as a new methodology in polymer synthesis. Several reviews have addressed this topic (Kobayashi, 2010; Yang et al., 2011; Yu et al., 2012; Zhang et al., 2013; Zhang et al., 2014), including extensive backgrounds on the lipase-catalyzed synthesis of polyesters from polyols and diacids.

Uyama et al. (1999) and Uyama et al. (2001) reported the lipase-catalyzed regioselective polymerization of divinyl sebacate and triols (glycerol-included). These studies produced acylglyceride products through the polymerization of divinyl sebacate and glycerol using *Candida antarctica* lipase as the catalyst. The reactions were performed at 60°C for 8 h with different reagent ratios. The obtained products were characterized by NMR and SEC analysis. A polymer yield of 63% was obtained in mass after washing. The main unit achieved was 1,3-diaclyglyceride with a small amount of the branching unit 1,2,3-triaclyglyceride. The reagent ratios greatly affected the microstructure of the polymer (molecular mass and glyceride distribution).

Choi and Yoon (2010) registered a patent for the preparation of a biodegradable polymer using an enzyme catalyst. Different sebacate-based polymers are mentioned, including PGS produced by enzyme B as a catalyst, in toluene medium.

Godinho et al. (2018) reported the successful synthesis of pPGS with *Candida antarctica* lipase B free (CALB) and lipase B immobilized Novozym 435 (N435) with crude glycerol, a by-product of biodiesel production, and glycerol. An equimolar G:S ratio was used, with the reactions performed at 60°C for 24 h in a *t*-butanol solvent. The products were characterized by MALDI-ToF-MS and NMR. The acid consumption (titration method) was around 75% for the immobilized enzyme and 68% for the free enzyme after 24 h. After rinsing with water, viscous liquid prepolymers were obtained at room temperature, consistent with the PGS map by Li et al. (2015). The MALDI analysis showed that the crude glycerol is favorable for producing cyclic structures, mainly with N435 as a catalyst. Although a clear explanation for this finding is lacking, it may be related to the interaction of NaCl (present in crude glycerol) with the formed oligomers and the enzyme catalytic center. The enzyme types showed differences in acid consumption, and N435 produced richer prepolymer in the range of longer oligomers. In general, multibranched (oligomer with more than one triglyceride structure) or hyperbranched (no free -OH groups in the oligomer) oligomers were not detected, and the 1,3-diaclyglyceride unit was the predominant structure. All prepolymers were mainly composed of low-mass oligomers (<1000 g mol^−1^), but tridecamers were also detected (<1600 g mol^−1^).

Perin and Felisberti (2020) used immobilized CALB to produce PGS in mild reaction conditions and studied the kinetics, chain growth, and branching behavior in different reaction conditions (solvents, temperatures, CALB amount, reagents feed ratio). These findings showed that, during the polycondensation reaction, CALB-catalyzed esterification and acyl migration occurred simultaneously. Thus, the PGS architecture changed from linear to branched throughout the progression of the reaction, with the branching resulting from the simultaneous CALB-catalyzed esterification and acyl migration. The different solvents strongly influenced the chain growth. The reactions performed in acetone, at temperatures ranging from 30°C to 50°C, had a higher molecular weight distribution (>10 kDa) compared to those for tetrahydrofuran, *t*-butanol, or acetonitrile (<3.5 kDa), under the same conditions. Contrary to the conventional method, the increase in temperature did not necessarily mean a faster reaction and higher molecular weight. In acetone, 40°C performed better than 50°C (Perin and Felisberti, 2020).

Avoiding solvents, some works adopted a hybrid way to produce pPGS using enzymatic synthesis. First, the prepolymerization mixture was heated to 120°C, under N_2_ protection, to form a homogenous transparent liquid mixture. After a 24 h reaction, the temperature was reduced to 90°C and N435 (around 10%–15% of the mass of the starting reagents) was added. The N_2_ atmosphere was then removed, and vacuum was applied progressively until the end of the reaction, which could take > 60 h (total time) (Lang et al., 2020; Ning et al., 2022). With these reaction procedures, Lang et al. (2020) produced PGS with Mn, Mw, and Đ values of 3700 g/mol, 63,900 g/mol, and 16.9, respectively, after 71 h.

More recently, Ning et al. (2022) demonstrated that N435 catalysis in bulk leads to higher molecular weight PGS compared to that for the conventional method. They also reached an acid consumption of 82% without the formation of a gel fraction, in equimolar reaction conditions, without solvents, at 90°C. The N435 catalysis restricted the interchain cross-linking relations, preventing the gel fraction products, and offered higher selectivity for the reaction of primary hydroxyl units. The N435-catalyzed synthesis enabled the preparation of PGS with Mn, Mw, and Đ values of 6000 g/mol, 59,400 g/mol, and 10 at 67 h, respectively. The authors also explored the application of non-solvents to enrich PGS in higher molecular weight chains by solvent fractionation, with methanol showing the best results.

The use of enzymatic catalysis avoids glycerol loss and significantly reduces the pPGS synthesis time compared to the conventional method. Enzymatic synthesis allows greater control in obtaining oligomers with a linear structure, leaving cross-linking for the curing step. This may be relevant for PGS modifications with alternative cross-linkers, such as acrylate or isocyanate moieties.

### 2.5 PGS polymer structure synthesis using other catalysts and reagents

PGS has been synthesized mainly by esterification reactions between glycerol and sebacic acid without catalysts. The previous section described some studies using enzymes as catalysts. Two of these studies replaced sebacic acid with divinyl sebacate (Uyama et al., 1999; Uyama et al., 2001). Several publications reported the use of other monomers and catalysts to obtain PGS structures. This section describes publications that used non-enzymatic catalysis and other monomers for PGS-type structure synthesis.

Organometallic catalysts are widely used in industry and research in the polymers field. Wyatt et al. (2012) used dibutyltin (IV)oxide as a catalyst to produce poly(glycerol-co-diacid)s, where sebacic acid was not selected. However, in a recent work, Wilson et al. (2018) used FASCAT 9100 (butylstannoic acid) catalyst to produce pPGS. After curing, a PGS with a tensile strength of 0.84 MPa was produced. In this work, a block copolymer of PGS with poly(tetramethylene oxide) glycol (PTMO) and a mixture of PGS-b-PTMO with a poly(ester-ether) thermoplastic elastomer (Hytrel 3078) was synthesized, producing a polymer, PGS-*b*-PTMO–Hytrel 3078, with extreme elasticity (2574% elongation).

Diarylborinic acid catalysts promote the formation of linear polyesters from glycerol. Slavko and Taylor (2017) used organoboron catalysts to produce polymers that were essentially free of branching or cross-linking. Sebacoyl chloride was used instead of sebacic acid, and PGS synthesis was performed in THF solvent at 70°C (Slavko and Taylor, 2017). Using sebacoyl chloride and glycerol as monomers and diarylborinic acids as catalysts, a high fraction of 1,3-diaclyglyceride units was found in NMR analysis (Slavko and Taylor, 2017). These findings demonstrated the production of PGS with an essentially linear structure.

Another alternative to PGS synthesis is the ring-opening reaction of diglycidyl sebacate with sebacic acid. Here, diglycidyl sebacate replaces glycerol. This approach aims to produce a linear PGS backbone. This reaction yielded a well-defined linear structure known as poly(sebacoyl diglyceride) PSeD, suitable for functionalization (You et al., 2010; Chen et al., 2016; Wang et al., 2016).

Wrzecionek et al. (2021) developed a method to obtain linear PGS by catalyst-free polytransesterification using glycerol and dimethyl sebacate (2:1 molar ratio, respectively). The authors fixed the molar ratio of the reactants and varied the time and temperature. The synthesis was optimized to minimize the degree of branching and maximize the molecular weight. The optimal parameters obtained for this process were 160°C and 30 h, which produced PGS with a branching degree of 3.5% and a molecular weight of 1.6 kDa.

### 2.6 PGS photopolymerization (acrylate cross-linking)

**FIGURE 6:**
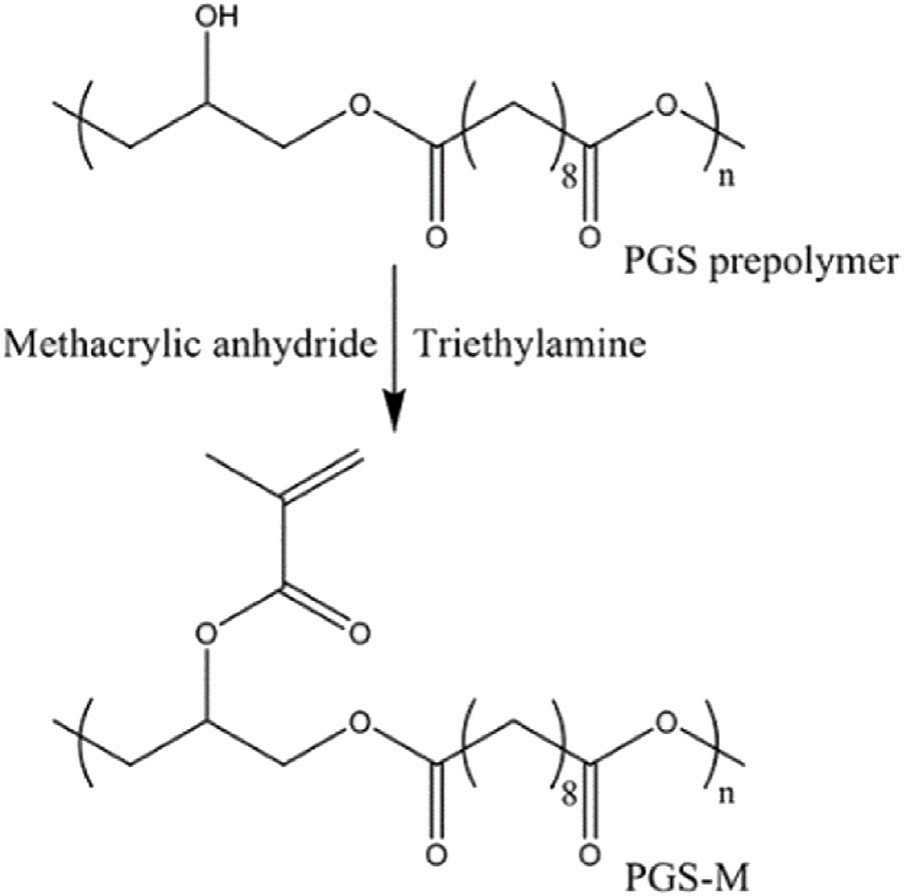
Synthesis schemes for PGS prepolymer and PGS-Methacrylate (PGS-M). Scheme from Pashneh-Tala et al. (2018).

The photopolymerization method has been used to obtain a final polymer by introducing reactive acrylate groups into pPGS to form PGS photocurable materials (Figure 6). This approach makes it possible to produce a wide range of physical properties under mild conditions using ultraviolet (UV) light photopolymerization and to reduce the curing step to a few minutes instead of days as in the traditional thermo-curing process. However, the preparation of these photocurable pPGS can also take a long time in functionalization reactions with acrylate groups (Nijst et al., 2007; Ifkovits et al., 2008; Mahdavi et al., 2008; Wu Y et al., 2014; Yeh et al., 2016; Wang M et al., 2017; Wang L et al., 2017; Hu et al., 2017; Yeh et al., 2017; Pashneh-Tala et al., 2018; Chen J.-Y et al., 2018; Singh et al., 2018; Farr et al., 2020; Kazemzadeh Farizhandi et al., 2020; Liang et al., 2020; Pashneh-Tala et al., 2020).

Nijst et al. (2007) synthesized PGS acrylate (PGSA) using acryloyl chloride and a photoinitiator (2,2-dimethoxy-2-phenylacetophenone). The synthesis of pPGS followed the traditional method and then was performed with the addition of acrylate moieties. The UV curing step required only 10 min. The elastomers showed Young’s modulus values of 0.05–1.38 MPa, an ultimate tensile strength of 0.05–0.50 MPa, and an elongation at break of 42–189%, depending on the degree of acrylation. Increasing acrylate led to an increased Young’s modulus and decreased elongation capacity. Photocured PGSA networks showed biocompatibility *in vitro* as assessed by human primary cell adherence and subsequent proliferation into a confluent monolayer. The copolymerization of poly(ethylene glycol) diacrylate with PGSA was also tested and allowed for additional control of final material properties.

Ifkovits et al. (2008) similarly synthesized PGSA as Nijst et al. (2007), with the same conclusions. In general, Young’s modulus increased with an increasing degree of acrylation. The elongation at break increased with increasing molecular weight for a constant degree of acrylation. In their study, the PGSA mechanical properties were 0.15–30 MPa (Young’s modulus) and 5%–200% (elongation). Not all macromers formed an elastomeric network. High acrylation values led to the formation of a very stiff PGSA with low elastomeric characteristics.

Acrylated and methacrylated PGS are biocompatible materials for use in biological tissue engineering applications. These types of materials have been proposed as aid materials for wound dressing (Mahdavi et al., 2008) and nerve guidance conduits (Hu et al., 2017; Singh et al., 2018).

Mahdavi et al. (2008) developed a synthetic gecko-inspired adhesive tissue in PGSA that may be useful for a range of medical applications, including sealing wounds and replacing sutures/staples.

**FIGURE 7:**
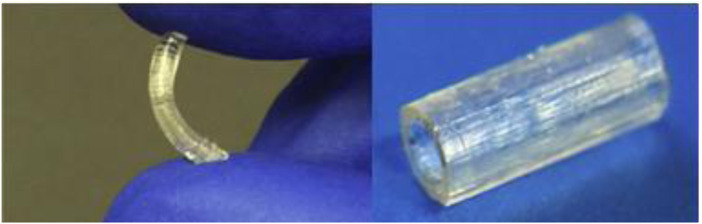
Methacrylated PGS nerve guidance conduits: the left is compressed to highlight the elastic properties, while the right shows the final 3D-printed product ready for implantation (Singh et al., 2018).

Methacrylated PGS has been proposed for nerve tissue applications (Hu et al., 2017; Singh et al., 2018). Because pPGS is a difficult material to electrospin into nanofibers, Hu et al. (2017) synthesized PGS-based copolymers with methyl methacrylate (MMA), a more easily processed material. Singh et al. (2018) used PGS methacrylate to produce nerve guidance conduits *via* stereolithography for peripheral nerve injury repair (Figure 7). The material showed appropriate mechanical properties and supported neuronal and glial cell growth *in vitro* and *in vivo*.

Yeh et al. (2016) reported the extrusion-based 3D printing of PGSA to produce scaffolds with elastic properties. This method showed great potential to originate complex biocompatible elastomeric tissues. In the same line of studies, the authors also developed a norbornene-modified PGS (Nor-PGS) that cross-linked faster under ultraviolet light (<1 min), suitable for extrusion-based 3D printing (Yeh et al., 2016).

Tsai et al. (2020) developed a new type of photocurable and elastomeric hydrogel using Nor-PGS-*co*-polyethylene glycol (Nor-PGS-co-PEG). The norbornene functional groups allowed hydrogel cross-linking *via* thiol-norbornene photochemistry. The cross-linking process was rapid in the presence of a photoinitiator and UV light (<3 min). Several properties of this material can be easily fine-tuned by adding different amounts of cross-linker. The Nor-PGS-co-PEG can be processed using electrospinning and 3D printing techniques to generate microfibrous scaffolds and printed structures, respectively. The material showed excellent elongation (around 950%) and good cytocompatibility in *in vitro* studies. Nor-PGS-co-PEG is a promising elastomer with highly tailorable properties for biomedical applications.

Chen J.-Y et al. (2018) described the tunable mechanical and degradation properties for the selection of biodegradable photocurable polymers that may be useful in 3D printing. The authors produced biodegradable photocurable copolymers by copolymerizing polycaprolactone diacrylate (PCLDA) and/or poly(ethylene glycol) diacrylate (PEGDA) with PGSA to form a polymer network. PCLDA and PEGDA are two common choices used in biomedical research. However, the degradation rates of these polymers *in vivo* are low, limiting their applications. The formation of several copolymers generated a database with selectable properties. The overall degradation rate was significantly higher than those for pure substances.

The direct use of PGS-based biomaterials in *in situ* tissue and cell encapsulation applications is limited due to their low water uptake. Therefore, Wu Y et al. (2014) developed injectable photocurable biodegradable hydrogels and microgels based on methacrylate poly(ethylene glycol)-co-poly(glycerol sebacate) copolymers. These gels showed good hydration properties and an easy *in situ* gelation process by photopolymerization under physiological conditions, thus demonstrating their potential as injectable tissue engineering scaffolds.

**FIGURE 8:**
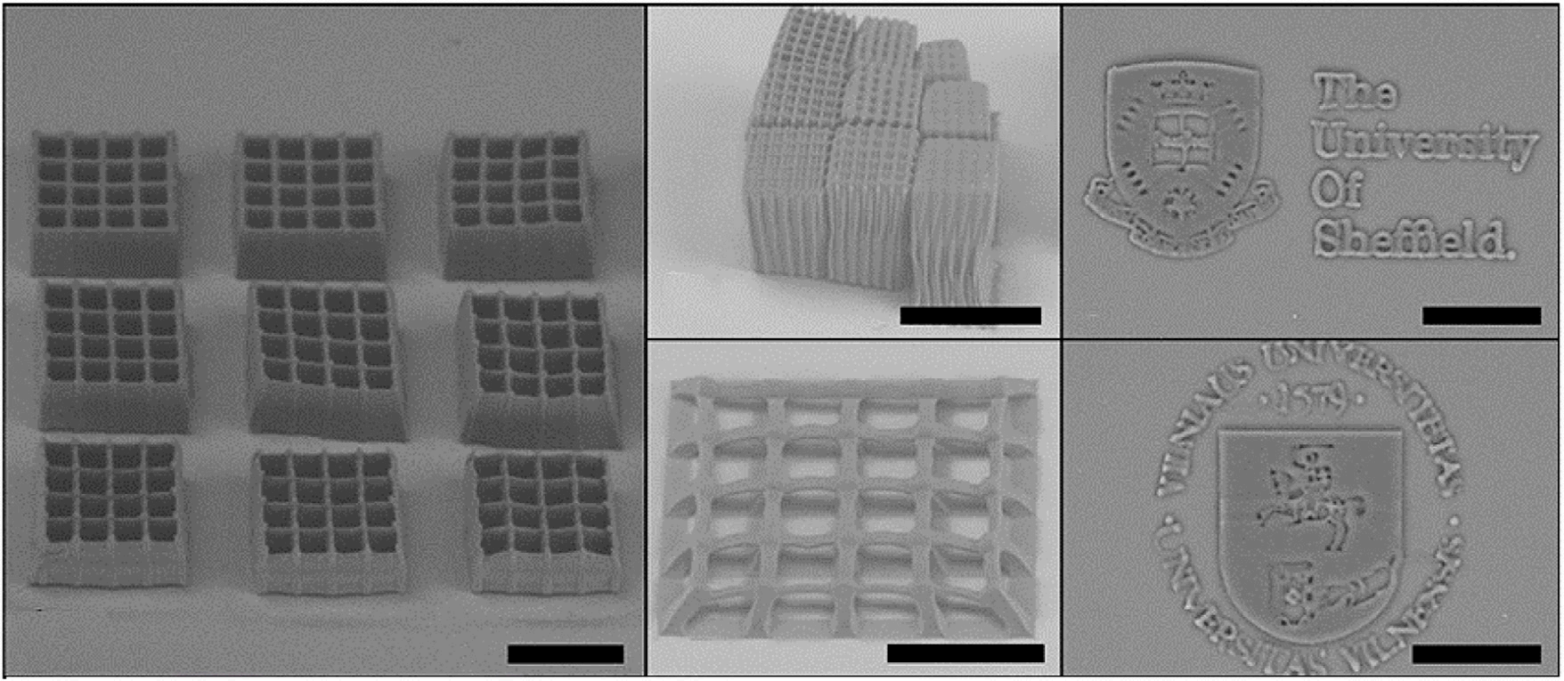
PGS-M 3D structures produced by DLW-2PP. Images collected and adapted from Pashneh-Tala et al. (2018).

Pashneh-Tala et al. (2018) and Pashneh-Tala et al. (2020) developed a photocurable PGS methacrylate (PGS-M) prepolymer by functionalization of secondary hydroxyl groups with methacrylic anhydride and triethylamine as catalyst. The authors used different approaches to define the shape of the final material. The authors filled molds with pPGS-M, applied UV light, and photopolymerized a generic disc shape to be CNC carved, creating different objects from digital designs with excellent manufacturing quality and a highly porous structure (Pashneh-Tala et al., 2020). The DLW-2PP (direct laser writing two-photon polymerization) laser technique to obtain 3D structures (Figure 8) was also used to produce PGS-M objects (Pashneh-Tala et al., 2018).

Wang M et al. (2017) produced a photo/thermo dual curable polymer based on PGS. The functionalization of PGS with 2-isocyanatoethyl methacrylate (IM) quickly produced a methacrylated PGS (PGS-IM). The PGS-IM was synthesized only by mixing PGS with IM at 80°C for 20 min in DMF solvent and without additional reagents/catalysts. After this process, PGS-IM scaffolds were produced by three curing approaches. The thermo-cured scaffolds used a vacuum oven at 150°C at 1 Torr for 12 h. The photo-cured scaffolds were produced using an Irgacure 2959 and UV light for 10 min. The dual-cured scaffold was produced by consecutively applying the previous two curing approaches. The photo-curing was applied first and then the thermo-curing. The combination of these curing processes provided a further way to modulate the properties of the resultant porous scaffolds. All PGS-IM scaffolds showed good elasticity, biodegradability, and cytocompatibility with L929 fibroblast cells. The cross-linking in PGS-IM comprised both acrylate and urethane bonds.

The next section describes the cross-linking of PGS by urethane bonds.

### 2.7 PGS urethane cross-linking

The use of isocyanates for pPGS cross-linking is another alternative to avoid the long curing times in the conventional process and to produce PGS-derived polymers with improved properties (Pereira et al., 2013; Li et al., 2015; Frydrych and Chen, 2017; Monem et al., 2022). The reaction between isocyanate and free hydroxyl groups occurs rapidly under mild conditions.

**FIGURE 9:**
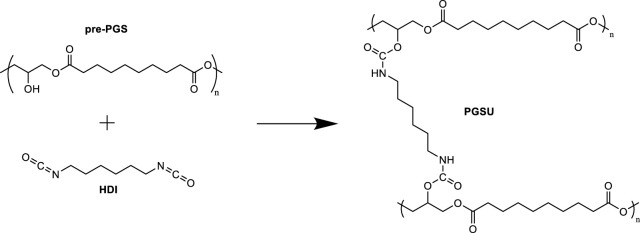
Reaction scheme for PGSU synthesis using HDI as a pre-PGS cross-linker. Scheme inspired by Pereira et al. (2013).

Pereira et al. (2013) produced a PGS urethane (PGSU) biocompatible and mechanically tunable elastomer suitable for encapsulation and controlled drug delivery systems. PGSU was synthesized with hexamethylene diisocyanate (HDI) as the cross-linker and tin (II) 2-ethylhexanoate as the catalyst (Figure 9). Pereira et al. (2013) synthesized PGSU films under two conditions: with solvent and solvent free. The solvent-free approach reduced the quantity of solvent traditionally used in film cast and produced films in <36 h. Pereira et al. (2013) reported a wide range of mechanical properties (Young’s modulus from 0.1 MPa to 20 MPa and elongations >400%) for their PGSU films, replicating the characteristics of some biological tissues. The *in vitro* assessment of the biodegradation and cytocompatibility demonstrated that the degradation profile depended on the degree of cross-linking. Increasing urethane content resulted in slower degradation rates. The degradation rates for all PGSU derivatives were generally slower than that for PGS. Testing of the cytocompatibility of the PGSU materials in human mesenchymal stem cells showed identical metabolism to cells placed in tissue culture polystyrene (TCP) after 8 days of cell proliferation. The inflammatory reaction *in vivo* of PGSU was significantly lower than that observed for poly(lactic-co-glycolic acid) (PLGA), a degradable material that has been FDA-approved for internal use.

Frydrych and Chen (2017) synthesized three-dimensional biodegradable PGSU scaffolds and films *via* solvent-based synthesis using HDI, tin (II) 2-ethylhexanoate, and 1,4-dioxane as solvent. The PGSU scaffolds showed good hydrophilic characteristics and high-water absorption abilities. *In vitro* tests, the PGSU scaffolds demonstrated variable degradation rates and mass losses of 10%–16% and 30%–62%, without and with the presence of lipase enzyme, respectively, after 112 days. The results demonstrated that the degradation kinetics of the PGSU scaffolds depended on the urethane content in the PGSU specimens, in which slower degradation rates were linked to higher urethane group numbers, and *vice versa*, similar to the findings reported by Pereira et al. (2013).

Frydrych et al. (2015b) produced polyester-based polyurethane (PEU) hydrogels based on PGS and poly(ethylene glycol)s (PEG)s. The hydrogels were thermoresponsive, stretchable, biodegradable, and biocompatible. The hydrogels had a tensile Young’s modulus, ultimate tensile strength, and elongation at break in the range of 0.02–0.20 MPa, 0.05–0.47 MPa, and 426%–623%, respectively. *In vitro* cell tests showed that some of the hydrogels were suitable for culturing adipose-derived stem cells and dermal fibroblasts. These results showed the versatility of these PEU hydrogels for biomedical and engineering applications.

Wang et al. (2018) synthesized PGSU and urethane-based PEGylated PGS elastomers using HDI, tin (II) 2- ethyl-hexanoate, and pPGS and pPEGS. These mixtures were allowed to react at 55°C with stirring for 5 h and under argon flow for cross-linking. By tailoring the PEG and HDI contents, elastomers were produced with broad ranges of mechanical properties and customized hydrophilicities. The mechanical properties of these elastomers are shown in Table 2. Increasing PEG decreased the water contact angle (WCA) to between 28.6–71.5°. The HDI amount had almost no influence on the hydrophobicity of polymers but influenced Young’s modulus and tensile strength. The degradation rate depended on the urethane content in the elastomers, as reported previously (Pereira et al., 2013; Frydrych and Chen, 2017). These elastomers showed favorable biocompatibility *in vitro* and mild host response *in vivo*. The results showed that these elastomers could be easily produced into various shapes and be tailored for diverse applications in biomedical research.

Monem et al. (2022) also synthesized PGSU using HDI, tin (II) 2-ethylhexanoate, and 1,4-dioxane as a solvent. In this study, a series of PGSU nanocomposites were synthesized and characterized to produce desirable elastomeric materials. These nanocomposites were prepared with two kinds of nanoclay under the commercial names of Cloisite Na^+^ and Cloisite 10 A. The results indicated that both nanoclays enhanced the storage modulus. Hydrolytic degradation of the nanocomposites indicated that the degradation behaviors of the samples were highly affected by their hydrophilicity properties. The neat PGSU showed a mass loss of 63.5 ± 1% after 30 days (degradation rate ∼15% per week) and a WCA close to 80°. The more resistant PGSU nanocomposite to degradation showed a mass loss of 48.9 ± 1% after 30 days (degradation rate ∼11% per week) and a WCA close to 90°.

Golbaten-Mofrad et al. (2021) similarly produced PGSU using HDI, tin (II) 2-ethylhexanoate, and a solvent mixture of DMSO:DMF (70:30 wt%). The solution was stirred at 55°C for 15 min. In this research experiment, a series of PGSU scaffolds with various cross-link densities were prepared for subsequent polypyrrole polymerization and insertion of zinc oxide (ZnO) nanoparticles. The mechanical performance of the scaffolds under dry and hydrated conditions was evaluated by compression tests. Hydrated low urethane content scaffolds presented Young’s modulus and compression stress at 75% strain in the ranges of 8.1–9.4 kPa and 26.6–29.8 kPa, respectively. In contrast, the high urethane content scaffolds displayed higher Young’s modulus values and compression stress at 75% strain in the ranges of 48.8–122.5 kPa and 927.9–1014.5 kPa, respectively. The ZnO nanoparticles improved the surface hydrophilicity (WCA 86°) and added anti-bacterial behavior (WCA 97.2°). The high HDI molar ratio intensified the samples’ surface hydrophobicity (WCA 102.9°).

Li et al. (2015) also synthesized PGSU, but with methylene diphenyl diisocyanate (MDI) as the cross-linker. They observed that MDI resulted in a more rigid polymer compared to PGS. Thus, isocyanate introduction must be moderate because excessive amounts remove the elastomeric properties of the final material.

## 3 PGS material properties

PGS is presently characterized as a material that resembles soft biological tissues. Its mechanical properties (Table 2) are close to those of some biological tissues, such as the cornea, the arteries/veins, the spinal cord, the gray matter, and some muscles (McKee et al., 2011). Because sebacic acid and glycerol both have endogenous natures, PGS and PGS-based materials are considered to be biocompatible (Piszko et al., 2021b). Moreover, glycerol and sebacic acid have been approved by the FDA; therefore, PGS degradation products are considered safe (Kemppainen and Hollister, 2010; Sha et al., 2021). PGS polyester elastomer can appear as transparent, almost odorless, and colorless or slightly yellow (depending on oxygen present during the reaction) (Halil Murat, 2017; Piszko et al., 2021b). PGS forms a covalently cross-linked 3D network of random coils with hydroxyl groups on the backbone (Sha et al., 2021). The PGS density is around 1.13 g/cm^3^ (Nagata et al., 1996; Pomerantseva et al., 2009).

pPGS is soluble in many available organic solvents, including 1,3-dioxolane, THF, dimethyl carbonate, ethanol, isopropanol, DMF, dioxane, acetic acid, formic acid, and acetone (Halil Murat, 2017; Piszko et al., 2021b). This makes processing easier and allows the use of a variety of techniques.

The physicochemical properties of PGS are commonly assessed by FTIR and NMR. These analyses are useful for screening the synthesis progress and characterizing the final material. FTIR confirms the presence of all important bonds and functional groups including polar hydroxyl, terminal carboxyl groups, ester bonding, and aliphatic backbone. NMR analysis allows for effective structural characterization of the prepolymer before subsequent cross-linking or modification, as well as analyses of the molecular chain topology (Wyatt et al., 2012; Li et al., 2013a; Halil Murat, 2017; Perin and Felisberti, 2020; Piszko et al., 2021a; Piszko et al., 2021b; Ning et al., 2022).

The thermal stability of PGS, as evaluated by thermal gravimetric analysis (TGA), is consistent throughout the literature. PGS is stable up to 250°C and shows a single weight loss step between 320°C and 475°C (Gaharwar et al., 2015; Tang et al., 2017; Aghajan et al., 2020; Rostamian et al., 2020; Martín-Cabezuelo et al., 2021a; Chang et al., 2021). The initial degradation temperature starts between 320°C and 350°C, with a peak degradation temperature typically between 435°C and 440°C (Gaharwar et al., 2015; Aghajan et al., 2020; Martín-Cabezuelo et al., 2021a; Piszko et al., 2021a). However, Martín-Cabezuelo et al. (2021a) studied the effects of the PGS synthesis under different atmospheres and observed a lower peak of thermal degradation (415°C and 425°C for hydrated and dry air, respectively) when PGS was synthesized using air (Martín-Cabezuelo et al., 2021a).

The thermal properties of PGS, as assessed by differential scanning calorimetry (DSC), are also consistent in the literature. PGS is a semi-crystalline polymer, with properties that depend on the glass transition temperature (*T*_*g*_) of the amorphous phase and melting temperature (*T*_*m*_) of the crystalline phase (Cai and Liu, 2007; Jaafar et al., 2010). The degree of crystallization decreases significantly with the extent of cure (Jaafar et al., 2010; Guo et al., 2014). The *T*_*g*_ of PGS ranged between −40°C and −15°C, with a broad melting transition between −20°C and 40°C according to the DSC diagrams (Cai and Liu, 2007; Jaafar et al., 2010; Conejero-García et al., 2017). PGS is completely amorphous >35°C (Cai and Liu, 2007; Jaafar et al., 2010; Rostamian et al., 2020). The dynamic mechanical thermal analysis (DMTA) results are consistent across many publications. The temperature at the maximum of the associated peak in *tan δ* shifts accordingly to lower temperatures, around -20°C, which characterizes the main relaxation process associated with the *T*_*g*_ of PGS (Aghajan et al., 2020; Rostamian et al., 2020; Martín-Cabezuelo et al., 2021a; Chang et al., 2021).

The crystallinity and morphology of PGS can be assessed by X-ray diffraction (XRD) analysis. PGS shows a broad amorphous peak at about 2θ = 20° which is related to the short-range regular ordered structure of both free and cured chains along with the disordered structure of the amorphous phase of the PGS matrix (Nagata et al., 1996; Nagata et al., 1999; Chen et al., 2007; Guo et al., 2014; Aghajan et al., 2020).

PGS is considered hydrophilic, with a WCA around 38–94° (Guo et al., 2014; Gaharwar et al., 2015; Aghajan et al., 2020; Chang et al., 2021; Martín-Cabezuelo et al., 2021a; Tevlek et al., 2022). A higher glycerol ratio synthesis promotes decreased WCA as it increases the number of hydroxyl groups. However, a higher ratio of sebacic acid increases the hydrophobic group content and WCA values (Guo et al., 2014). For PGS produced from a molar reagent ratio of 1:1 (G:S), the increase in cross-link density, which consumes more hydroxyl groups, provides more wettable surfaces (Conejero-García et al., 2017; Tevlek et al., 2022). However, when the cross-link density increases by urethane (Golbaten-Mofrad et al., 2021) or methacrylate (Singh et al., 2018) bonds, the WCA value increases (Singh et al., 2018; Golbaten-Mofrad et al., 2021). This can be contradictory. However, the hydrophilicity of PGS elastomers is related not only to the presence of hydroxyl groups but also to the polar end groups and inter-molecular hydrogen bonds (Conejero-García et al., 2017; Tevlek et al., 2022). The WCA value of a PGS material is a good indicator of cell viability as more wettable surfaces promote better cell adhesion and propagation (Fakhari et al., 2021; Tevlek et al., 2022).

Based on the ISO 10,993-5 standard, materials with cell viability <70% are considered toxic. PGS can cause cytotoxicity *in vitro* due to acidic components released into the culture medium because of surface degradation (Li et al., 2013b; Tevlek et al., 2022). PGS elastomers with lower cross-link density degrade faster than those with higher cross-link density, in the same environmental conditions. The unreacted carboxylic acid groups and/or the carboxylic acids produced by the hydrolysis of ester groups can cause severe acidification of the medium (Li et al., 2013b), leading to higher cytotoxicity of PGS elastomers with lower cross-link density (Chen et al., 2011; Li et al., 2013b; Tevlek et al., 2022). Moreover, Liu et al. (2009) reported high cytotoxicity of a PGS elastomer modified with citric acid (PGSC). After 7 days, the accumulated acidity of the acidic sols inhibited the growth of L-929 cells, causing most of the cells to die (Liu et al., 2009). These findings confirmed that excessive acidity caused by elastomer degradation leads to high cytotoxicity levels.

However, the addition of another acid monomer to the polymer structure does not necessarily imply increased cytotoxicity. Chen et al. (2011) reported that the addition of lactic acid to PGS to obtain (PGS-co-LA) significantly improved the cytocompatibility of the final materials compared to the PGS alone.

PGS and PGS-based materials are non-toxic when synthesized properly. The cytocompatibility of PGS has been demonstrated in NIH 3T3 fibroblasts (Wang et al., 2002), 3T3 fibroblasts (Rai et al., 2013a; Jeffries et al., 2015), MC3T3 osteoblasts (Wu et al., 2016), chondrocytes (Wu et al., 2016), human umbilical artery smooth muscle cells (HUASMCs) (You et al., 2016; Hsu et al., 2018; Wu H. J et al., 2019), SNL mouse fibroblasts (Chen et al., 2011; Li et al., 2013b; Xu et al., 2015), L-929 fibroblasts, (Conejero-García et al., 2017; Varshosaz et al., 2021; Wang et al., 2021; Jia et al., 2016; Tang et al., 2017; Wu et al., 2016; Wang M et al., 2017) human umbilical vein endothelial cells (HUVECs) (Wang et al., 2021; Zhang et al., 2021; Tevlek et al., 2022), hFOB1.19 human fetal osteoblasts (cytocompatibility and osteoconductivity) (Piszko et al., 2021a), Schwann cells (Sundback et al., 2005; Singh et al., 2018), bone marrow stromal cells (BMSCs) (Wang et al., 2018), human mesenchymal stem cells (hMSCs) (Pereira et al., 2013), and human dermal fibroblasts (Pashneh-Tala et al., 2020).

PGS and PGS-based material biocompatibility has been demonstrated *in vivo* in BALB/c adult mice (Piszko et al., 2021a), CD^®^ (Sprague-Dawley) IGS rats (Fu et al., 2020b), Sprague-Dawley rats (Wang et al., 2002; Ifkovits et al., 2008; Jia et al., 2016; Ma et al., 2016; Wu et al., 2016; Xiao et al., 2019), Wistar rats (Mahdavi et al., 2008; Sun et al., 2009), Fisher rats (Sundback et al., 2005), rabbits (osteoconductive to bone regeneration) (Zaky et al., 2017), C57 rats (Wang et al., 2018), YFP+ mice (Singh et al., 2018), and Lewis rats (Pomerantseva et al., 2009; Pereira et al., 2013). *In vivo*, some mild and temporary inflammatory responses typical of implantable biodegradable polymers have been reported with PGS and PGS-based materials; however, necrosis or tissue degradation have not been reported (Ifkovits et al., 2008; Pomerantseva et al., 2009; Sun et al., 2009; Wu et al., 2016; Fu et al., 2020b).

Table 2 presents the mechanical properties of different PGS-based materials found in the bibliography. The data shows how compliant PGS can be with other elements, allowing the production of new materials with different or improved mechanical properties.

### 3.1 PGS degradation (*in vitro* hydrolytic, *in vitro* enzymatic, and *in vivo*)

PGS degradation can be evaluated by three methods: *in vitro* hydrolysis degradation, *in vitro* enzymatic degradation, and *in vivo* degradation. Independent of the degradation type, the PGS degradation process follows the surface erosion mechanism (Wang et al., 2003; Sundback et al., 2005; Pomerantseva et al., 2009; Guo et al., 2014; Kim et al., 2014; Li et al., 2015; Souza et al., 2017; Zhang et al., 2020; Tevlek et al., 2022). This mechanism is characterized by linear mass loss and corresponding volume decrease while preserving the shape, surface integrity, and mechanical properties. The surface erosion mechanism has also been observed in many PGS-based materials such as PGSU (Pereira et al., 2013; Frydrych and Chen, 2017), PGS-M (Pashneh-Tala et al., 2018), and other PGS-based materials (Ma et al., 2016; Lang et al., 2020).

However, Shi et al. (2020) demonstrated that induced cracks overcome erosion in PGS and lead to the premature loss of the mechanical properties and morphology of the material. The crack progression depends on pH, humidity, and applied forces (Shi et al., 2020).

The degradation rate, for the same conditions, is related to the cross-link density of PGS materials, in which materials with higher cross-link densities show more resistance to degradation (Pereira et al., 2013; Li et al., 2015; Frydrych and Chen, 2017; Lau et al., 2017; Singh et al., 2018; Krook et al., 2020).

Krook et al. (2020) investigated the degradation of porous PGS, reporting that the polymer properties change rapidly with degradation in the case of materials with lower cross-link density.

#### 3.1.1 *In vitro* hydrolytic degradation

The *in vitro* hydrolytic degradation is typically performed in a buffered aqueous solution (pH 7.4), at 37°C under agitation. This type of degradation is the slowest.

One study reported that the PGS samples lost 15%–30% of the mass, depending on the cross-link density, during the 28-day process of hydrolytic degradation (Li et al., 2015). In another study, the PGS slowly degraded, losing only 17% of the mass in 60 days (Sundback et al., 2005).

In another study, PGS-IM scaffolds degraded *in vitro* showed mass losses of 12.2% (photo-cured), 11.9% (thermo-cured), and 5.9% (dual-cured), respectively, at day 28. The dual-cured scaffolds showed the lowest mass loss rate, likely due to their highest cross-link density (Wang M et al., 2017). Once again, this process of degradation is slower.

#### 3.1.2 *In vitro* enzymatic degradation

The *in vitro* enzymatic degradation is usually performed in an aqueous buffered solution (pH 7.4), with enzymes (e.g., lipases and esterases), at 37°C, and under agitation. The use of enzymes accelerates the degradation process.

Nagata et al. (1996) performed the *in vitro* degradation of PGS films with lipase. After 6 h, a weight loss of 80 g/m^2^ was observed for PGS films, with a reaction degree of 83%. The degree of reaction affected the degradation. PGS films with higher degrees of reaction showed higher resistance to degradation.

Nagata et al. (1999) performed *in vitro* degradation of PGS copolymers films with lipase. The films were obtained by incorporating other diacids in the polymer synthesis. The various PGS copolymers films produced were compared after 2 h of enzymatic degradation. The PGS film had a weight loss of 50 g/m^2^; however, the addition of other diacids increased the resistance of the copolymer films. For example, replacing 10% (mol) of sebacic acid with succinic acid resulted in a PGS-co-succinate film, “Yg-10/4 (90/10)”, which had a weight loss of 22 g/m^2^, an increase in degradation resistance of >50%, compared to PGS.

Tevlek et al. (2022) cured three sets of PGS elastomers for different times (14, 12, and 10 h) and performed *in vitro* hydrolytic and enzymatic degradations in a 28-day process. The hydrolytic mass losses were 9.65%, 13.79%, and 24.82%, and the enzymatic degradation mass losses were 12.75%, 19.54%, and 43.75%, respectively. The cross-link densities were 70.33, 33.79, and 14.77 mol/m^3^, respectively. Their data confirmed that enzymatic degradation was faster than hydrolytic degradation and that the degradation rate of both depended on the cross-link density.

In a 4-day process, Pereira et al. (2013) performed *in vitro* enzymatic degradation in PGS and PGSU with different degrees of urethane cross-linking. The PGS samples were completely degraded in 4 days, while the mass was progressively lost in the PGSU samples due to their cross-link density. The PGSU samples with the highest cross-link densities showed 0% mass loss (no degradation).

Frydrych and Chen (2017) performed *in vitro* degradation tests in PGSU scaffolds, which showed adjustable degradation rates and mass losses of 8.7%–16.3% and 10.7%–20.7% without and with the presence of enzyme, respectively, after 31 days. Enzymatic degradation was faster than hydrolytic degradation.

Singh et al. (2018) performed PGS-M hydrolytic degradation studies after 40 days, in which the implants showed no change in mass (no degradation). Enzymatic degradation results indicated a decrease in the degradation rate of the polymer with an increased degree of methacrylation. The results of enzymatic degradation showed a decreased rate of polymer degradation with an increased degree of methacrylation. At the highest methacrylation cross-linking the degradation rate was null (no degradation).

The information presented thus far in this review showed that the modification of PGS can lead to significantly increased resistance to degradation.

#### 3.1.3 *In vivo* degradation

*In vivo* degradation is performed by placing the object to degrade inside an animal (e.g., mice or rats). In *in vivo* trials, the environment is more dynamic, with a more fluid exchange of molecules and removal of any degradation products around the implant. The presence of various enzymes in their natural environment also has a greater impact on degradation, compared to *in vitro* trials (Ifkovits et al., 2008; Pomerantseva et al., 2009). The *in vivo* degradation rate of PGS is much faster than the *in vitro* degradation rate.

Wang et al. (2002) demonstrated the differences between *in vitro* and the *in vivo* PGS degradation. In the 60-day trials, the measured *in vitro* hydrolytic degradation of PGS resulted in a 17.6% mass loss, while PGS implanted in Sprague-Dawley rats were completely consumed in the same time.

Wang et al. (2003) implanted PGS samples subcutaneously in female Sprague-Dawley rats and evaluated them after 35 days. The PGS implants maintained their geometries throughout the time periods. The implants lost weight gradually and linearly over the test period of 35 days, during which time >70% of their mass.

Another study implanted PGS samples in male Fisher rats, after which the degradation was evaluated for 60 days. After 35 days, the geometry of the PGS implants was the same as that on day 1; however, the volume was almost half that measured initially. The implants gradually decreased in size, consistent with a mechanism of surface erosion. After 60 days, the implants were difficult to detect and no dimensional data were obtained (Sundback et al., 2005).

The PGS was almost completely degraded within 14 days in the arterial circulation of Sprague-Dawley IGS rats (Fu et al., 2020a).

Ding et al. (2017) added tyramine (TA) to PGS and placed the PGS-TA and PGS in male BALB/cJ mice. After 14 days, both implants had completely degraded *in vivo*.

The rapid degradation of PGS may limit its use in tissues that require long-term mechanical support but may be useful for the controlled release of drugs in short-term treatments.

PGS implants loaded with 5-fluorouracil (5-FU-PGS) placed in Wistar rats maintained their geometries and decreased in bulk throughout the degradation period of 30 days. The mass loss *in vivo* (30%) was much higher than that *in vitro* (10%). The results of the *in vitro* anti-tumor activity assay suggested the anti-tumor activity of 5-FU-PGSs exhibited through sustained drug release. These results showed that PGS is a good candidate for drug delivery systems (Sun et al., 2009).

The rapid hydrolysis of PGS limits its application as a scaffold material in tissue engineering applications, particularly when healing is slow (i.e., from months to years) (Lang et al., 2020).

However, PGS showed good results for guided tissue regeneration. Upon implanting PGS in the rabbit ulnar defect, histology and tomography analysis at 8 weeks showed that gap filling with the new bone, guided by the PGS elastomer (Zaky et al., 2017).

Another way to use PGS for long-term treatment is by mixing it with other components.

PGS combined with chondroitinase ABC (ChABC) promoted spinal cord repair in rats in 12 weeks. The combination of PGS and ChABC resulted in augmented nerve regeneration and partial functional recovery, better than PGS or ChABC independently (Pan et al., 2018). A recent study used PGS scaffolds to restore a wounded rat uterus, which promoted BMSC attachment and growth and increased blood vessel regeneration in 90 days (Xiao et al., 2019).

PGS modification by functionalization of the hydroxyl groups with palmitates (palmitate-PGS) has been successfully shown to delay degradation (Fu et al., 2020b; Ding et al., 2020). *In vivo* tests with CD^®^ (Sprague-Dawley) IGS rats showed that palmitate-PGS degraded over 4–12 weeks compared to only 2 weeks for PGS alone (Fu et al., 2020b).

PGSA samples were implanted in Sprague-Dawley rats. After 4 weeks, the *in vivo* mass loss (25%) was greater than *in vitro* hydrolytic (12%). Past 8 weeks, the *in vivo* mass loss (37%) was nearly the same at the *in vitro* hydrolytic mass loss (33%) (Ifkovits et al., 2008).

## 4 Outlook/Conclusion

PGS is an elastomer-type polymer with great potential in the biomedical field because its biocompatibility and properties can be tailored to biological tissues. PGS is typically produced through the polycondensation of glycerol and sebacic acid. However, its synthesis has also been reported using divinyl sebacate or sebacoyl chloride with glycerol. Another alternative method of producing PGS is by ring-opening reaction of diglycidyl sebacate with sebacic acid, which results in a well-defined linear structure known as PSeD.

PGS was mainly produced by the conventional method, which is energy-intensive and time-consuming without the use of solvents or catalysts. One strategy to reduce the reaction time is increasing the temperature to >150°C. However, this option can lead to a significant loss of glycerol and an increased number of branches and/or cross-links in the polymer chain and, thus, a more rigid material.

MwAS reportedly produces pPGS in minutes instead of hours or days and is mainly mixed with other materials. Microwave radiation promotes the growth of undifferentiated polymers, in which the primary and secondary hydroxyl groups have the same reactivity with carboxylic acid groups. This type of pre-PGS is richer in cross-linked structures (triacylglycerides) and requires less time to cure.

The use of catalysts is the least often described approach to potentially reduce reaction time. Enzymes and diarylborinic acids can be used to reduce the reaction time and temperature by promoting linear structures. However, the use of solvents requires polymer separation and purification steps.

Another strategy to reduce the PGS synthesis time is the modification of pPGS with cross-linkers such as isocyanates and acrylates that speed curing. Isocyanates allow fast cross-linking *via* urethane bonds, while acrylate and methacrylate allow fast cross-linking by photopolymerization.

PGS has been frequently combined with other molecules and polymers to produce materials with more desirable properties. Individually, PGS has a high biodegradability *in vivo* that is not suitable for long-term applications. However, its biocompatibility and safety are well proven, which makes PGS a valid polymer for the development of materials for biomedical applications.
